# HPV Integration Site Mapping: A Rapid Method of Viral Integration Site (VIS) Analysis and Visualization Using Automated Workflows in CLC Microbial Genomics

**DOI:** 10.3390/ijms23158132

**Published:** 2022-07-23

**Authors:** Jane Shen-Gunther, Hong Cai, Yufeng Wang

**Affiliations:** 1 Gynecologic Oncology & Clinical Investigation, Department of Clinical Investigation, Brooke Army Medical Center, Fort Sam Houston, TX 78234, USA; 2 Department of Molecular Microbiology and Immunology, University of Texas at San Antonio, San Antonio, TX 78249, USA; hong.cai@utsa.edu; 3 South Texas Center for Emerging Infectious Diseases, University of Texas at San Antonio, San Antonio, TX 78249, USA

**Keywords:** bioinformatics workflow, cervical cancer, HPV taxonomy, human papillomavirus, hybrid capture NGS, insertional mutagenesis, next generation sequencing, viral mapping, virus integration

## Abstract

Human papillomavirus (HPV) integration within the host genome may contribute to carcinogenesis through various disruptive mechanisms. With next-generation sequencing (NGS), identification of viral and host genomic breakpoints and chimeric sequences are now possible. However, a simple, streamlined bioinformatics workflow has been non-existent until recently. Here, we tested two new, automated workflows in CLC Microbial Genomics, i.e., Viral Hybrid Capture (VHC) Data Analysis and Viral Integration Site (VIS) Identification for software performance and efficiency. The workflows embedded with HPV and human reference genomes were used to analyze a publicly available NGS dataset derived from pre- and cancerous HPV+ cervical cytology of 21 Gabonese women. The VHC and VIS workflow median runtimes were 19 and 7 min per sample, respectively. The VIS dynamic graphical outputs included read mappings, virus-host genomic breakpoints, and virus-host integration circular plots. Key findings, including disrupted and nearby genes, were summarized in an auto-generated report. Overall, the VHC and VIS workflows proved to be a rapid and accurate means of localizing viral-host integration site(s) and identifying disrupted and neighboring human genes. Applying HPV VIS-mapping to pre- or invasive tumors will advance our understanding of viral oncogenesis and facilitate the discovery of prognostic biomarkers and therapeutic targets.

## 1. Introduction

Human papillomavirus (HPV) is the most common viral cause of cancer worldwide [1,2]. In 2020, the global estimate for new HPV-attributable cervical cancer cases was 604,000 and 120,000 for other anogenital and oropharyngeal sites [2,3,4]. The cause of cervical cancer remained elusive until 1983, when zur Hausen and his team made the breakthrough discovery of isolating HPV-16 and -18 from cervical and other genital cancers, and later finding viral DNA integrated within the host genome [5,6]. Over the last 40 years, incremental research has revealed how HPV replicates within host cells and integrates fortuitously to mediate malignant transformation [7]. An efficient means of localizing viral-host integration site(s) will advance our understanding of carcinogenesis and facilitate the discovery of prognostic biomarkers and therapeutic targets.

The HPV genome is an ~8000 base pair (bp), double-stranded, circular DNA encoding 6 early genes (*E1*, *E2*, *E4*, *E5*, *E6*, and *E7*) and 2 late genes (*L1* and *L2*) [8]. Throughout the viral life cycle, HPV replicates in three distinct phases, namely, “initial replication” after viral entry, “maintenance replication” during cellular replication, and “vegetative replication” in differentiated cells [9]. “Accidental integration” may result from intimate interactions between the viral and host genomes throughout replication [10]. During cell division, two models of viral genome replication have been described, i.e., Random Attachment and Faithful Partitioning [10,11]. Specifically, the HPV genome in the form of extrachromosomal plasmids may tether randomly or pairwise to sister chromatids at the viral origin of replication (ori) by HPV E2 binding. Next, the viral genome unwinds bidirectionally at the ori via the HPV E1 helicase, replicates using cellular proteins, and partitions equally into the daughter cells [9,10,11,12]. However, during vegetative amplification, HPV E1/E2 loads onto the viral ori and binds directly to host chromatin to initiate, recruit, and exploit cellular DNA damage response for self-replication [10].

HPV integrates fortuitously but preferentially at vulnerable regions of the host genome. Chromosomal fragile sites (loci sensitive to replication stress and breakage) and transcriptionally active regions of open chromatin are reportedly more common [7,9]. Post integration, the HPV *E2* gene (often with *E1*) is frequently disrupted and functionally incapacitated for *E6*/*E7* repression. One study identified the *E2* hinge region (3172 to 3659 bp) as the most frequently disrupted site; the most prevalent site for *E1* was located between 1059 and 1323 bp [13]. Partial or complete *E1*/*E2* gene loss may also occur. The rates of HPV-16 *E1* and *E2* disruption between benign and malignant cervical tumors are also significantly different (9% vs. 46%) and (18% vs. 63%), respectively [14]. Yet the mechanism behind preferential disruption remains unclear [15]. The HPV integrant, invariably composed of the *E6* and *E7* oncogenes, is consequently unleashed to drive tumorigenesis [9]. As for the integration site, host cancer-associated genes and/or neighboring genes may be disrupted and/or functionally altered leading to malignant transformation [12].

The era of next generation sequencing (NGS) has transformed viral discovery and metagenomic research. Viral enrichment strategies have also been developed and employed to obtain greater depth of coverage for regions of interest and comprehensive variant profiling. The two most common approaches for target enrichment are amplicon sequencing and hybrid-capture NGS [16,17]. For the latter, targets (whole genomes or regions of interest) are captured by hybridization to target-specific biotinylated probes, isolated by magnetic pulldown, and sequenced [16,17]. The distinctive benefits of this method include identification and mapping of virus-host chimeric reads and virus/host genomic breakpoints for integration site localization. A variety of kits are now commercially available for hybrid-capture NGS, e.g., SureSelect Target Enrichment (Agilent, Santa Clara, CA, USA), DNA prep with Enrichment (Illumina, San Diego, CA, USA), KAPA Target Enrichment (Roche, Wilmington, MA, USA), and QIAseq xHYB (QIAGEN, Germantown, MD, USA) [18,19,20,21]. Contrarily, the accompanying bioinformatics analysis has been lagging in development and implementation. Both manual and developer-based, viral-host mapping workflows, i.e., SearcHPV, and nf-VIF (nextflow-based Virus Insertion Finder) have been reported [22,23,24]. However, drawbacks of open-source software must be considered by the inexpert user to include: (1) prerequisite for advanced computational skills, (2) uncertainty of software maintenance and support (“abandonware”), (3) security risk, and (4) prohibition by some governmental institutions [25,26,27]. In 2021, CLC Microbial Genomics developed two automated workflows, i.e., Viral Hybrid Capture (VHC) data analysis and Viral Integration Site (VIS) identification dedicated to viral investigations [28]. The user-friendly, GUI-based, commercial software has minimized the obstacles mentioned above.

Here, we tested the performance of the pre-built VHC and VIS workflows used in tandem for HPV integration analytics. We leveraged curated HPV and human reference genomes (easily accessible within CLC) by embedding them within the workflows to reduce computation time and ambiguous results. Our findings demonstrate that VHC/VIS workflows offer a rapid and accurate means of localizing viral-host integration site(s) for identifying disrupted and neighboring host genes of clinical significance.

## 2. Results

### 2.1. Viral Hybrid Capture (VHC) Analysis and Visualization

The entire dataset comprised of 21 FASTQ files and 2.9 GB of digital information was downloaded in 08:31 min. The cytological categories are provided in Section 4.1. The VHC workflow median runtime per sample was 18.5 min (range, 6.5 to 37 min). The QC workflow generated the following outputs: (1) QC for sequencing reads (graphical report and supplementary report), and (2) Abundance table. Specifically, the graphical report summarized the total number of sequences and nucleotides in a sample, per-sequence analysis, per-base analysis, over-representation analyses, sequence duplication levels, and duplicated sequences. The QC supplementary report includes two additional columns, i.e., “coverage” and “abs” for absolute numbers of sequences or bases for the per-sequence or per-base analyses. The reader is referred to the CLC MGM manual online for an in-depth explanation of QC metrics [28].

#### 2.1.1. HPV Taxonomic Profiling

**Figure 1:**
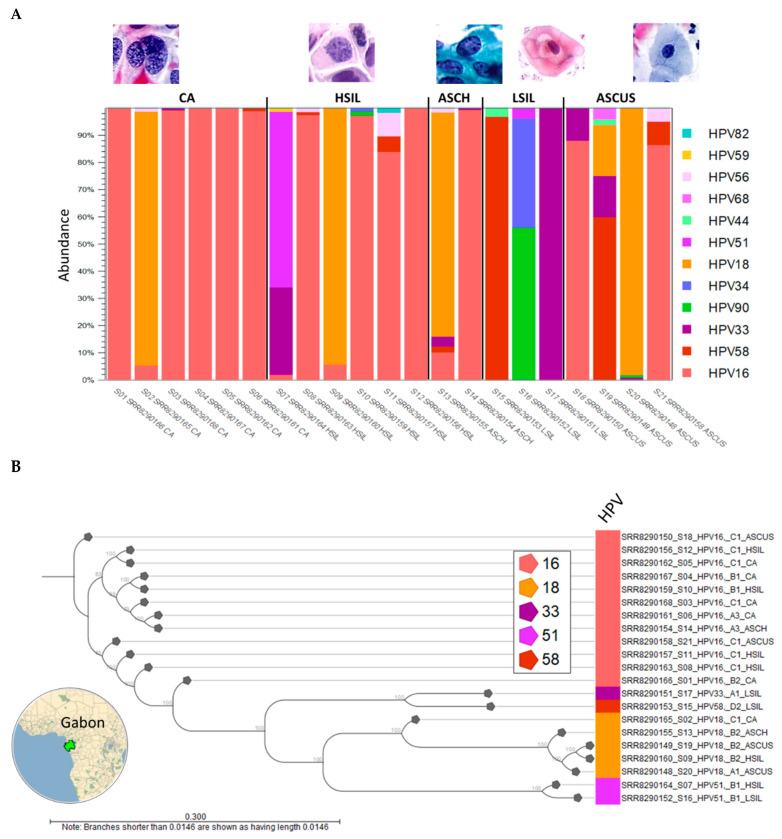
Viral Hybrid Capture (VHC) analysis. (**A**) Relative abundance of HPV genotypes found in individual samples (*n* = 21) after taxonomic profiling are shown as stacked bars. The total number of unique HPV genotypes identified in the cohort was 12. HPV type-specific carcinogenicity (carcinogenic, possibly carcinogenic, and not carcinogenic) are colorized in shades of red, blue, and green, respectively (legend); (**B**) The dominant HPV genotype and sub-lineage (alphanumeric) of each sample was genetically distinct with divergent branches in the phylogenetic tree. For the 15 HPV-integrated samples, the dominant and integrated genotypes were identical for 13 samples. Conversely, for S16 and S19, the dominant genotypes were HPV-90 and HPV-58, while the integrated types were HPV-90 and -51 (in tree), and HPV-18 (in tree), respectively. This finding suggests that type-specific insertional potential may prevail over viral counts for an integration event. Representative cytological images (40×) (source: IARC Cytopathology of the uterine cervix—digital atlas [30]).

The HPV taxonomic profiling workflow produced individual abundance tables that displays the names of the identified taxa, 7-level taxonomic nomenclature, coverage estimate, and abundance value (raw or relative number of reads found in the sample associated with the taxon). Low abundance genotypes were cut-off at a threshold of <1% of total composition. The merged abundance table (Supplementary Table S1) lists all taxonomic profiling results and the summary statistics, e.g., combined abundance of reads for the taxon across all samples, and the minimum, maximum, mean, median, and standard deviation of the number of reads for the taxa across all samples. The graphical output of the merged abundance table is shown as a stacked bar chart in Figure 1A. HPV type-specific carcinogenicity (carcinogenic, probably/possibly carcinogenic, and not classifiable/carcinogenic) are colorized respectively in shades of red, blue, and green in Figure 1A. HPV carcinogenicity was grouped as follows: (1) carcinogenic—HPV types 16, 18, 31, 33, 35, 39, 45, 51, 52, 56, 58, and 59, (2) probably carcinogenic—HPV type 68, (3) possibly carcinogenic—HPV types 26, 30, 34, 53, 66, 67, 69, 70, 73, 82, 85, and 97, (4) not classifiable—HPV types 6, 11, and (5) probably not carcinogenic—all other HPV types [29].Visualization as a sunburst plot may be achieved with 1-click (not shown).

#### 2.1.2. HPV Sublineages and Phylogenetic Tree

To determine the HPV sub-lineage of the dominant genotype within each sample, the “HPV consensus sequence” generated from the VHC workflow was aligned against the “HPV VAR” BLAST database using the CLC BLAST tool. The BLAST output table is provided in Supplementary Table S2. The “HPV VAR” BLAST database was created from the “HPV VAR” sequence list, as described in Section 4.2, using the “Create BLAST Database” tool.

To construct the phylogenetic tree based on HPV genotype and sub-lineages, the “HPV consensus sequence” output from the VHC workflow of the 21 samples were aligned collectively and analyzed phylogenetically using the “Create Alignment” and “Create Tree” tools sequentially. The resulting neighbor-joining (NJ) tree was labeled according to sample ID, sample number, HPV genotype, sub-lineage, and cytological grade as shown in Figure 1B. A map of Gabon was constructed using Wolfram Mathematica 13.0 (Champaign, IL, USA).

For our dataset, the HPV-16 sub-lineages of 12 samples were easily identified by BLAST as predominantly African ~83% (10/12): A3 (*n* = 2), B1–2 (*n* = 3), and C1 (*n* = 7) (Figure 1B). Identification of HPV-16 sub-lineages and variants is of clinical importance in terms of oncogenic risk and vaccine efficacy. A recent global study of HPV-16 sub-lineages (A, B, C, and D) showed how regional specificity (e.g., A3–4 East Asia; B1–4 and C1–4 Africa) may influence cervical cancer risk [31].

#### 2.1.3. Viral Hybrid Capture (VHC) Tracklists

**Figure 2:**
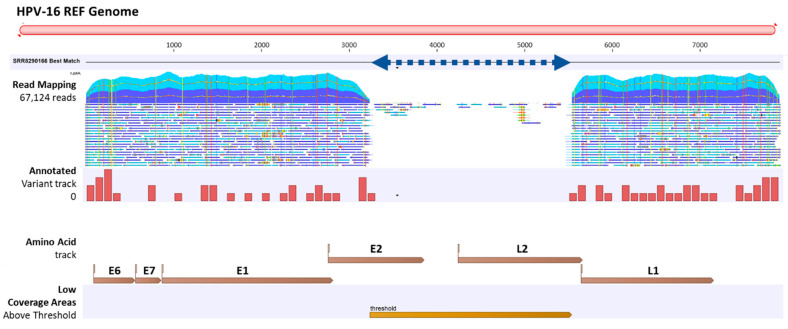
Viral Hybrid Capture (VHC) track list view. A representative VHC track list (S01) displays (top to bottom): read mapping against the HPV-16 reference genome, annotated variant track, HPV amino acid track, and low coverage areas. Low coverage region corresponds to the HPV-16 genomic gap from 3300 to 5550 bp (blue dashed line). For the read mapping track, only the top portion is shown. Scrolling down the entire length of the read mapping revealed a persistent *E2* to *L2* break indicative of a completely integrated form, whereas an intact full-length mapping would denote an episomal form.

The VHC workflow generated a “Track List” containing: (1) read mapping track, (2) annotated variant track, (3) amino acid track, and (4) low coverage areas track. A representative track list (S01) shows paired-reads mapped onto the linearized HPV-16 reference genome (Figure 2). The large gap between reads (blue dashed line) is automatically detected in the “low coverage areas” track using the default threshold criteria (best match reference genome coverage < 30×). The annotated variant track shows low frequency variants detected using the default threshold (coverage > 30× and frequency ≥ 20%). Finally, the amino acid track shows the virus-coded amino acids generated from the coding DNA sequence (CDS) annotation of the HPV REF sequence list chosen as the “Best Reference for Read Mapping” in the workflow. Zooming in on the track list to the nucleotide or amino acid level allows for detailed comparison to the reference genome and detection of variants (not shown). The track lists for all samples (S01 to S21) are provided in Supplementary Figure S1.

### 2.2. Viral Integration Site (VIS) Analysis and Visualization

**Figure 3:**
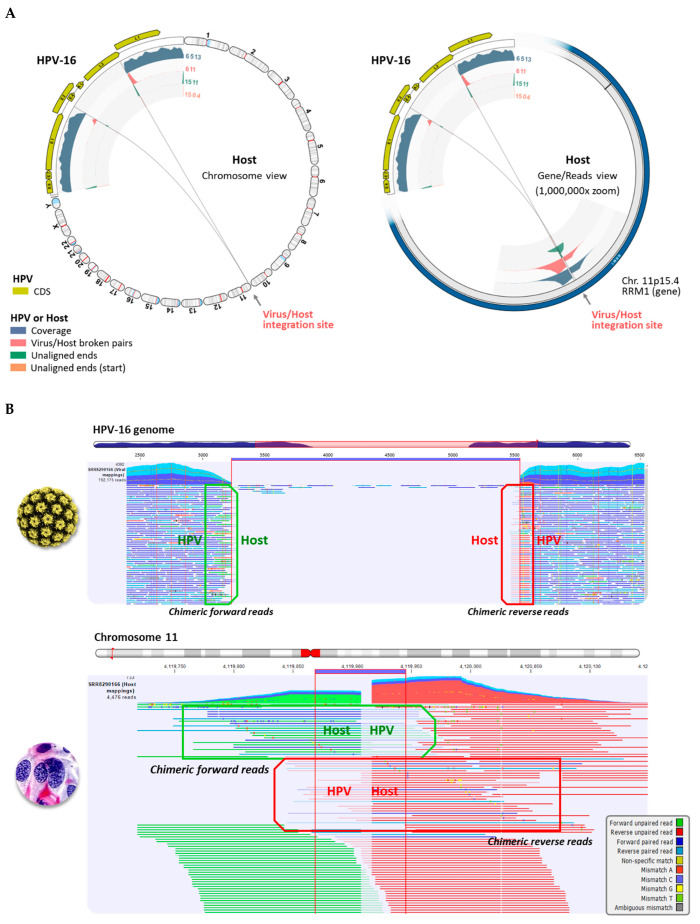
Viral-host Integration Site (VIS) Analysis (**A**) VIS circular plots in chromosome view (left) and gene view (1,000,000× zoom, right) for sample S01; (**B**) Read mapping to HPV-16 and chromosome 11 at sites of broken read-pairs and gaps (thick blue line) reveal forward and reverse viral-host chimeric reads. The mappings are truncated due to the extensive length.

**Figure 4:**
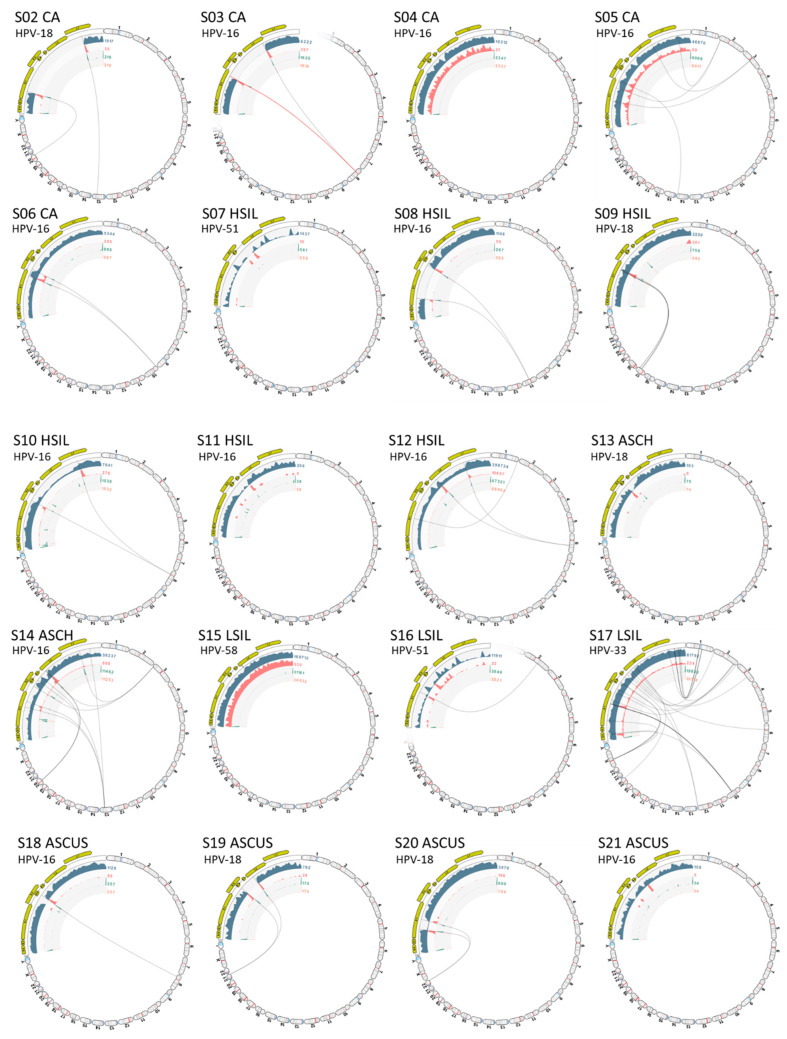
Viral Integration Site (VIS) circular plots. Collage of VIS circular plots for samples (S02 to S21) in chromosome view. Virus-host integration linkages manifested as chimeric reads are designated by the bi-directional curvilinear lines. For S16, HPV-90 and -51 were integrated in chromosomes 7 and 2, respectively (only the HPV-51/chr. 2 plot is shown). The dynamic functions (rotate and zoom) of the VIS circular plot are presented in Supplementary Video S1.

The VIS workflow median runtime per sample was 6.6 min (range 3.0 to 30 min). The workflow generated the following output files: (1) viral mapping and breakpoints annotation track, (2) host mapping and breakpoints annotation track, (3) zoomable and rotatable VIS circular plot, and (4) VIS summary report. A representative VIS circular plot (S01) presents the entire HPV and human genome in a circular layout with inner circles of different read tracks (Figure 3A). Virus-host integration linkages, i.e., chimeric reads are shown as bi-directional curvilinear lines, and read coverage (color-coded histogram tracks) are mapped onto genome coordinates (Figure 3A). For S01, a large gap in the HPV genome between *E2* and *L2* genes was easily discernable, and viral-host integration within chromosome 11 was detected. The 1,000,000× gene-level view exposes the host integration site(s), disrupted gene, and nearby genes. The dynamic functions of the VIS circular plot and read mapping tracks are presented in a brief video (Supplementary Video S1). In the HPV and host read mappings for S01, sites of broken read-pairs and viral-host chimeric forward/reverse reads are displayed and magnifiable to the nucleotide level for detailed inspection (Figure 3B). A collage of VIS circular plots for S02 through S21 is presented in Figure 4. The HPV genome gaps or low coverage areas are easily identified by the read histograms (gray) along with integration sites within the host genome. Additionally, HPV genotype and cytological grade may be applied as metadata for grouping and comparative analysis of integration sites. Finally, the auto-generated summary reports with tables of disrupted and nearby genes for all samples are provided in Supplementary Table S3.

HPV integration was detected in 15 of the 21 samples (Figure 4). For each sample, the number of chromosomes with integrated HPV DNA ranged from 1 to 8 as follows: 1 (60%), 2 (20%), 3 (13%), and 8 (7%). Remarkably, one outlier (S17 LSIL) harboring HPV-33 integrated with 8 chromosomes. As for the 23 (22 and X) chromosomes, the rate of integration events per chromosome by the 15 samples were similar: 0 (33%), 1 (29%), 2 (21%), and 3 (17%). Due to the small sample size, no trend analysis was performed. However, a systematic review of 25 studies analyzing 192 integration sites revealed a random distribution of integration events over the entire genome [7]. Notably, a predilection for fragile sites was identified, and the highest number of integration events befell upon chromosome 8q24 [7].

### 2.3. Workflow Runtimes

**Figure 5:**
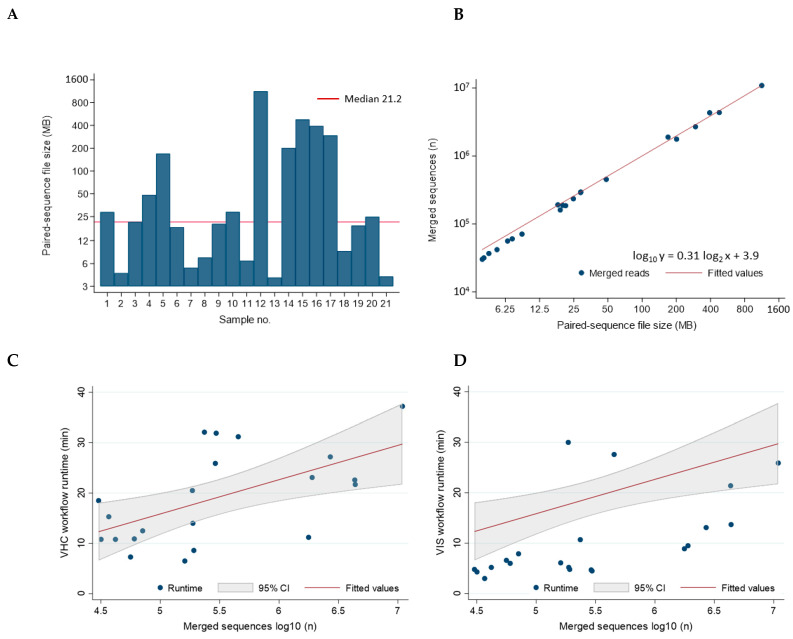
Correlation between NGS Reads and VHC/VIS Workflow Runtimes. (**A**) The sequencing file sizes of the 21 samples ranged broadly between 3.91 and 1130 MB with a median of 21.2 MB; (**B**) The file size correlated near-perfectly with the number of merged sequences after log_2_–log_10_ transformation, respectively (R^2^ = 0.998); (**C**,**D**) The number of merged reads (log_10_) correlated positively with VHC and VIS workflow runtimes in a linear-log relationship. The correlation was modest for both VHC and VIS with R^2^ = 0.335 and R^2^ = 0.316, respectively. The regression equations may be utilized for estimation of workflow runtimes based on number of merged reads. Log transformation was performed to compress the wide range of X- or Y-values making it suitable for linear modeling.

The sequencing file size of the 21 samples ranged broadly between 3.91 and 1130 MB with a median of 21.2 MB consisting of 186,028 merged reads (Figure 5A). The file size correlated near-perfectly with the number of merged sequences after log_2_–log_10_ transformation (Figure 5B). The median runtime per sample for the VHC and VIS workflows were 18.5 min (range, 6.5 to 37 min) and 6.6 min (range 3.0 to 30 min), respectively. The combined VHC/VIS median runtime per sample was only 23.3 min (range 12.6 to 63 min). These timed results not only demonstrated workflow efficiency but established a benchmark for future studies. A modest correlation between number of merged sequences/sample and VHC and VIS runtimes was found with R^2^ = 0.335 and R^2^ = 0.316, respectively (Figure 5C,D). In practice, the regression equations derived from the correlation analysis may be utilized for estimating runtimes based on the number of merged reads/sample (Figure 5C,D). Statistical analyses were performed using STATA/IC 17.0 (StataCorp LP, College Station, TX, USA).

## 3. Discussion

In this study, we tested the VHC and VIS workflows for software performance and efficiency using a deep sequenced, clinical dataset. By incorporating curated HPV and human genome databases within CLC MGM workflows, we were able to execute independent computational processes simply by inputting the data, selecting the reference genomes, and setting the parameters. Taxonomic classification and visualization of HPV metagenomes were accomplished efficiently and quickly to reveal compositional differences between samples. The VHC mapping and track list with zoomable visualization provided effortless inspection of mapped regions, variants, and low coverage areas at the nucleotide and amino acid levels. The median processing time for the VHC workflow was only 19 min/sample using a laptop computer. Furthermore, the HPV consensus sequences derived from the VHC workflow was valuable for elucidating the HPV sub-lineages and evolutionary relationships between samples. Similarly, the VIS workflow processed the NGS data swiftly with a median runtime of 7 min/sample. The autogenerated VIS outputs included viral and host breakpoint annotations tracks, a zoomable and rotatable VIS circular plot, and a summary report of disrupted and surrounding genes. The dynamic circular plot displayed the virus/host breakpoints and integration sites with just a few computer-mouse scrolls. The organized summary report facilitated review and identification of pathogenic genetic alterations.

In comparison to Nkili-Meyong and colleagues’ work, the VHC and VIS workflows unified numerous manual steps and automated time-consuming read mappings against viral and human references [22]. The incorporation of curated Human and HPV reference and variant genomes offered greater accuracy and efficiency in genotyping and variant identification then uncurated GenBank genomes. Finally, the auto-generated tables, reports, and visualizations from the workflows abolished the shortcomings of manual data processing, i.e., time, cost, and inadvertent errors. Of note, differences in search parameters (e.g., 100 vs. 500 KB for nearby gene distance) accounted for expected discrepant results. As mentioned in the Section 1, developer-based workflows have also been reported for the advancement of viral integration site analysis [23,24]. However, the drawbacks of command-line based, open-source software must be deliberated. Instead, simple workflows within a GUI-based platform offer a pragmatic solution for inexpert practitioners of bioinformatics, e.g., graduate students, clinical virologists, and physician-scientists.

Nkili-Meyong et al. found an increased rate of HPV integration as cytopathology worsened: ASCUS 30.8% (4/13), LSIL 60% (3/5), ASCH 66.7% (2/3), HSIL 71.4% (5/7), and Carcinoma 85.7% (6/7) [22]. Normal cytology (HPV-positive and -negative), however, was not included as a control group. Inclusion of the Negative for Intraepithelial Lesion or Malignancy (NILM) category is warranted in future studies to establish a baseline integration rate.

Beyond cervical cancer, Harle et al. reported the use of hybrid-capture NGS for confirming a tongue metastasis from a primary HPV16+, stage T3NxM0 anal cancer by matching HPV insertional signatures [32]. McEllin et al. similarly used hybrid-capture NGS for detecting HPV in the brain metastases of two patients with primary oropharyngeal squamous cell carcinoma (OPSCC) [33]. Both tumors contained HPV-16 that had integrated within chromosome 8q24.21 or 14q24.1 (known HPV integration hotspots) [33]. Most importantly, these cases illustrate the clinical utility of NGS and VIS mapping for proper diagnosis, treatment, and surveillance of cryptic, virally integrated tumors.

We acknowledge that our study has limitations that is, only one clinical dataset derived from custom SeqCap EZ probes was used for performance testing. Commercial kits produced by different manufacturers should be tested comparatively in the current “sequencing/computing” paradigm of viral research. To bridge this gap, we intend to start with a new, standardized hybrid-capture NGS kit (QIAseq xHYB Viral STI Panel) dedicated to detecting and genotyping 19 high-risk HPV genotypes, HBV, and HIV-1 [21]. Detecting HPV genotypes and variants beyond the panel will necessitate the design of a custom kit. A strength of the hybrid-capture NGS technology is that the starting material may be genomic RNA or DNA extracted from cells, fresh or formalin-fixed paraffin-embedded (FFPE) tissues collected from various sources [21]. Circulating tumor DNA (ctDNA)/NGS may also be considered for mutational profiling since it has shown promise in detecting recurrent head and neck cancers prior to clinical relapse [34]. Furthermore, single-cell RNA sequencing (scRNA-seq) for transcriptomic analysis may be used concurrently to dissect the heterogeneity of a mixed tumor cell population. Recent studies have shown that HPV-associated cancers may contain multiple viral integration sites, but often a singular, dominant “driver” site is active transcriptionally [15]. Therefore, HPV integration site mapping and transcriptomic analysis are promising, complementary molecular techniques applicable to targeted, individualized therapy.

## 4. Materials and Methods

### 4.1. NGS Dataset of HPV-Positive Cytology Samples

We used a publicly available dataset of 21 HPV-positive, liquid-based cytology specimens from a Gabonese population generously deposited by Nkili-Meyong et al. [22]. The cervical cytological grades of the samples were: Carcinoma (*n* = 6), HSIL (*n* = 6), ASCH (*n* = 2), LSIL (*n* = 3), and ASCUS (*n* = 4). After cellular DNA extraction, the DNA genomic library of this cohort were constructed using a double-capture, probe-based technique (SeqCap EZ probes) to enrich for HPV fragments prior to sequencing on the Miseq instrument [22]. The Gabon dataset is available from the European Nucleotide Archive (ENA) (https://www.ebi.ac.uk/ena) (accessed on 20 August 2021) under Accession Number: SUB4880803 [35]. For this study, the raw sequencing files and metadata were directly downloaded into CLC Genomics workbench using the “Search for Reads in SRA” tool under Sequence Read Archive (SRA) Accession Number: SRA819683 for downstream analysis. The Run Accession numbers, SRR8290148 to SRR8290168 and corresponding sample numbers listed in the metadata file are used for this study. Sample numbers (01 to 21) were abbreviated alphanumerically as “S01” to “S21.” The dataset was imported into the VHC/VIS workflows for viral integration mapping and visualization.

### 4.2. Customized HPV Reference Database and Human Reference Genome for CLC Workflows and Tools

**Figure 6:**
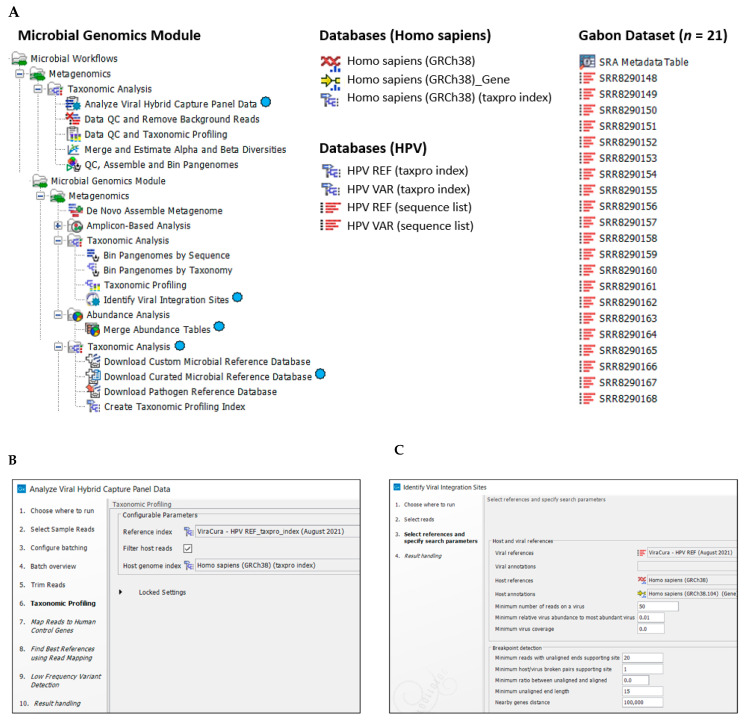
Bioinformatics methods (**A**) CLC Microbial Genomics Module, databases and dataset used for Viral Hybrid Capture (VHC) data analysis and Viral Integration Site (VIS) analysis. Primary workflows and tools used for this study are designated by the virus icon (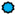); (**B**) VHC workflow steps (1–10) with user-defined parameter settings for Taxonomic Profiling (bold), i.e., HPV reference index and Host genome index; (**C**) VIS workflow steps (1–4) with selected HPV and Host reference genomes and user-defined search parameters entered for this study.

Customized HPV reference (*n* = 219) and variant (*n* = 139) genomes were downloaded using the “Download Curated Microbial Reference Database” tool built within the CLC Microbial Genomics Module [28]. Two formats (taxonomic profiling index and sequence list) were downloaded and incorporated into the VHC and VIS workflows (Figure 6A). The names of the databases in index and list formats, respectively, were: (1) “HPV REF_taxpro_index” and “HPV REF” for HPV reference genomes, and (2) “HPV VAR_taxpro_index” and “HPV VAR” for HPV variant genomes. For HPV reference genomes, the taxonomic nomenclature was annotated to the “genotype” level [36].

For HPV variant genomes, the taxonomic nomenclature was annotated to the “sub-lineage” level [36]. HPV genotypes, variants, and sub-lineages have >10%, 1.0%, and 0.5% to 1.0% nucleotide sequence difference of the HPV L1 open reading frame (ORF). However, nomenclature based on the entire HPV genomic sequence has been proposed recently by the International Committee on Taxonomy of Viruses (ICTV) [37]. The human reference genome Homo sapiens-Genome Reference Consortium Human Build 38 (GRCh38 or hg38) files were downloaded using the “Download Genomes from Public Repositories” function within the CLC Microbial Genomics Module (Figure 6A).

### 4.3. Viral Hybrid Capture Analysis and Workflow

CLC Genomics Workbench 21.0.4 and CLC Microbial Genomics Module (CLC MGM) 21.0 (Redwood City, CA, USA) were installed on an HP notebook computer (specifications: Windows 10 operating system, Intel i7-7500U dual-core processor @ 2.70 GHz and 8 GB RAM) for all analyses. The CLC system requirements are provided online [38]. The “Analyze Viral Hybrid Capture (VHC) Data” ready-to-use workflow of CLC MGM was used for automated data analysis (Figure 6A,B). The analysis consisted of 4 primary steps: (1) Data import, (2) Data quality control (QC), (3) Taxonomic Profiling of reads mapping to HPV and human reference genomes, and (4) Low frequency variant detection (Figure 6B). Post-workflow output included tables and visualization tracks for read mapping, annotated genetic variants, annotated amino acids, and low coverage areas.

### 4.4. Viral Integration Site (VIS) Analysis and Workflow

The “Identify Viral Integration Sites (VIS)” ready-to-use workflow of CLC MGM was used for automated data analysis (Figure 6A,C). The analysis consisted of 4 primary steps: (1) Data import, (2) Reads mapping to human and HPV reference genomes, (3) Breakpoint detection in human and viral genomes, and (4) Gene identification surrounding breakpoint(s) (Figure 6C). Workflow outputs included tables, read mapping to host and HPV genomes, and circular plot of viral-host genomes zoomable from the chromosome to gene level.

## 5. Conclusions

The CLC VHC and VIS workflows embedded with HPV and human reference genomes proved to be a rapid and efficient method of HPV taxonomic profiling, read mapping, and integration site analysis. The streamlined workflows will undoubtedly accelerate genomic exploration and advance our understanding of viral carcinogenesis.

## Data Availability

The Gabon dataset is available from the European Nucleotide Archive (ENA) (https://www.ebi.ac.uk/ena, accessed on 20 August 2021) under Accession Number: SUB4880803.

## Supplementary Materials

The following are available online at https://www.mdpi.com/article/10.3390/ijms23158132/s1. Reference [39] is cited in the Supplementary Table S2.

## Abbreviations

ASCUS, atypical squamous cells of undetermined significance; ASCH, atypical squamous cells, cannot exclude HSIL; BLAST, Basic local alignment search tool; chr, chromosome; ctDNA, circulating tumor DNA; CLC MGM, CLC Microbial Genomics Module; ENA, European Nucleotide Archive; GB, gigabyte; GUI, graphical user interface; HPV, Human papillomavirus; HSIL, high-grade squamous intraepithelial lesion; IARC, International Agency for Research on Cancer; KB, kilobyte; LSIL, low-grade squamous intraepithelial lesion; MB, megabyte; NGS, next-generation sequencing; NILM, negative for intraepithelial lesion or malignancy; QC, quality control; SCC, squamous cell carcinoma; scRNA-seq, single-cell RNA sequencing; VHC, Viral Hybrid Capture; VIS, Viral Integration Site.
