# Supplementary material for: HPV Integration Site Mapping: A Rapid Method of Viral Integration Site (VIS) Analysis and Visualization Using Automated Workflows in CLC Microbial Genomics
Source: Int J Mol Sci. 2022 Jul 23;23(15):8132. doi: 10.3390/ijms23158132 (PMC9331699; doi:10.3390/ijms23158132)
Supplement: Supplementary file 1 [file ijms-23-08132-s001.zip › FIG S1 VHC TRACKLISTS.pdf]

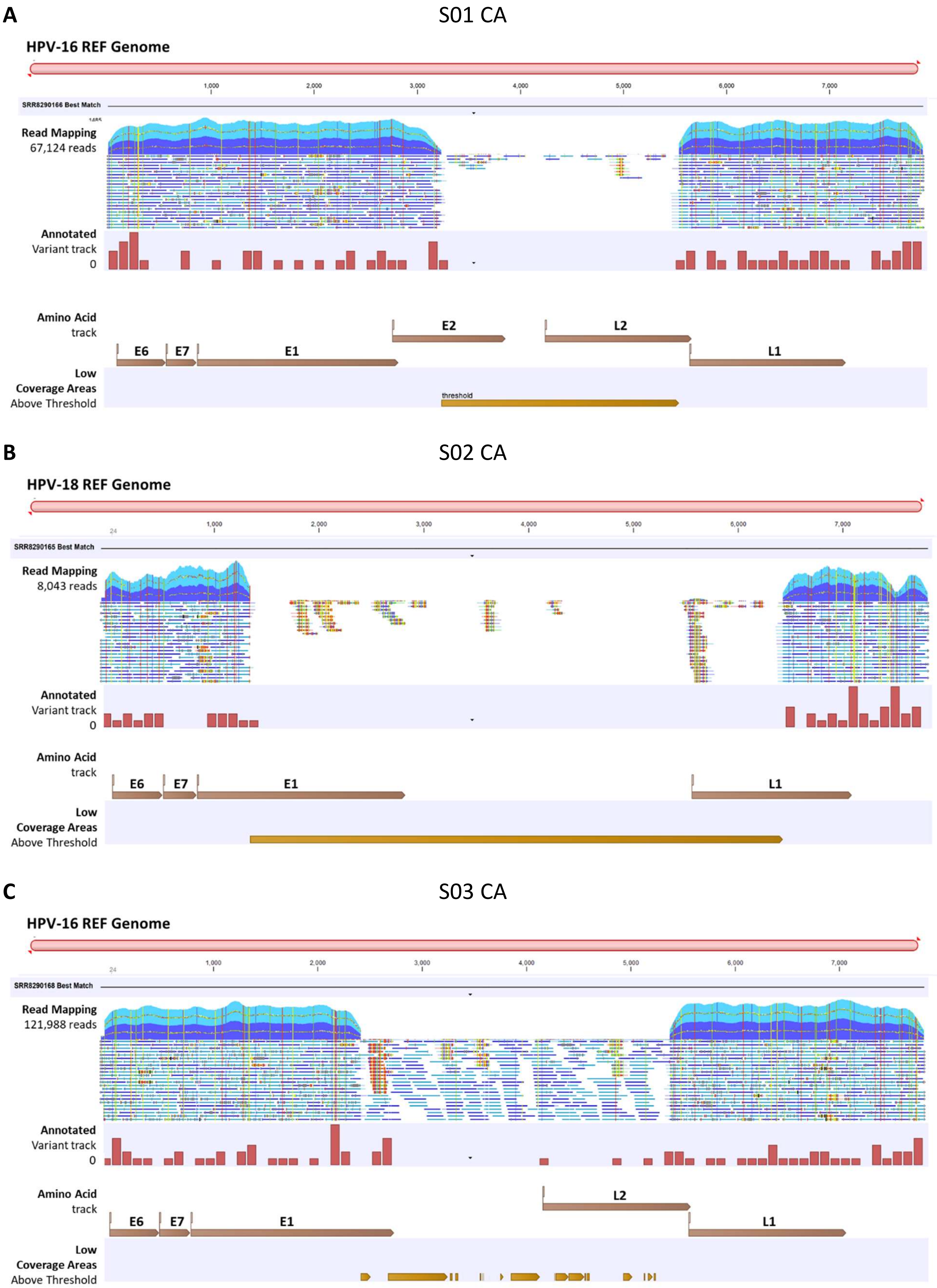
A
S01 CA
HPV-16 REF Genome
1,000
2,000
3,000
4,000
5,000
6,000
7,000
SRR8290166 Best Match
Read Mapping
67,124 reads
Annotated
Variant track
0
Amino Acid
track
E6
E7
E1
E2
L2
L1
Low
Coverage Areas
Above Threshold
threshold
B
S02 CA
HPV-18 REF Genome
24
1,000
2,000
3,000
4,000
5,000
6,000
7,000
SRR8290165 Best Match
Read Mapping
8,043 reads
Annotated
Variant track
0
Amino Acid
track
E6
E7
E1
L1
Low
Coverage Areas
Above Threshold
C
S03 CA
HPV-16 REF Genome
24
1,000
2,000
3,000
4,000
5,000
6,000
7,000
SRR8290168 Best Match
Read Mapping
121,988 reads
Annotated
Variant track
0
Amino Acid
track
L2
E6
E7
E1
L1
Low
Coverage Areas
Above Threshold

D

S04 CA

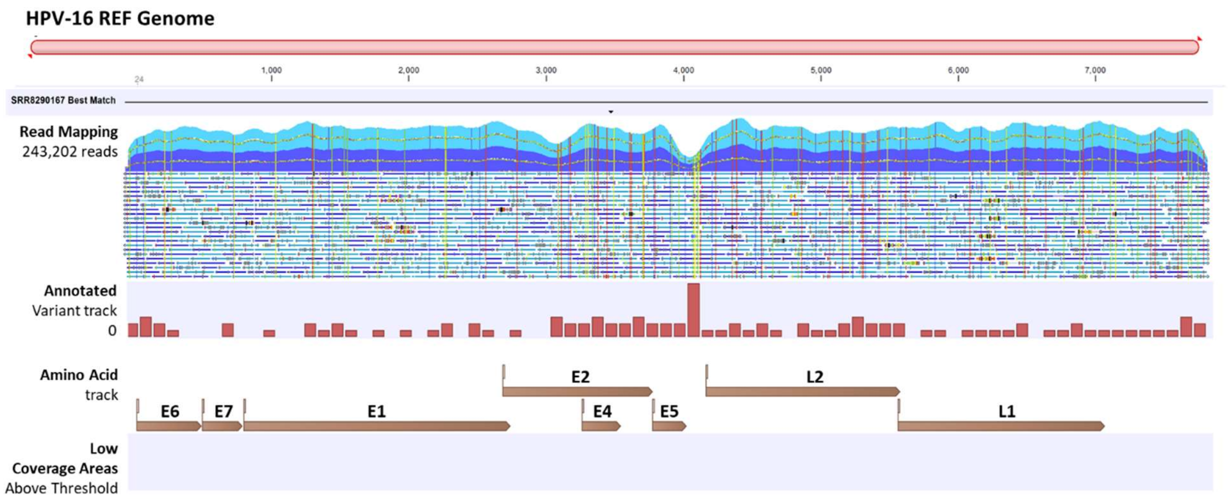

E

S05 CA

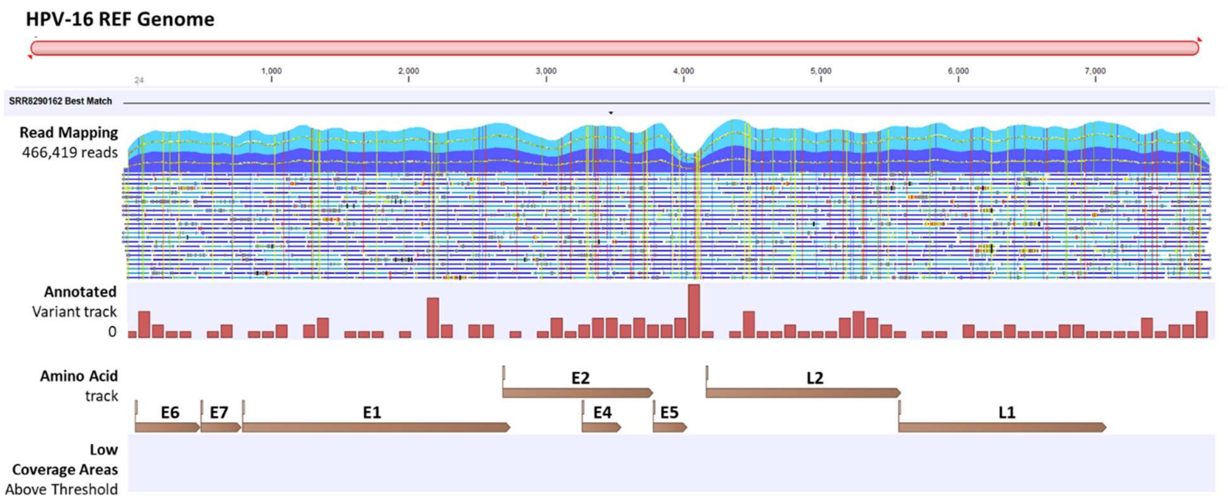

F

S06 CA

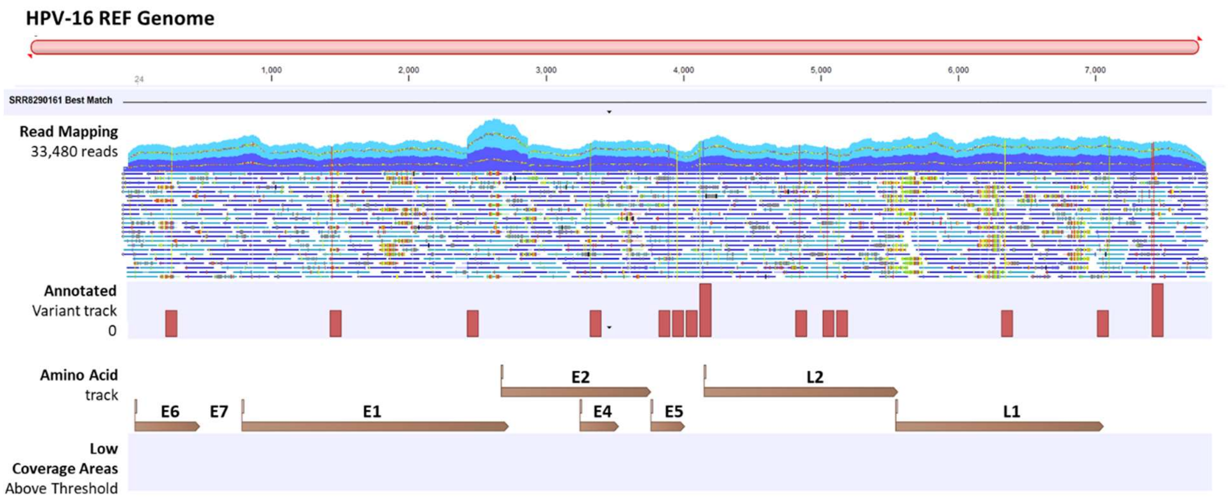

G S07 HSIL

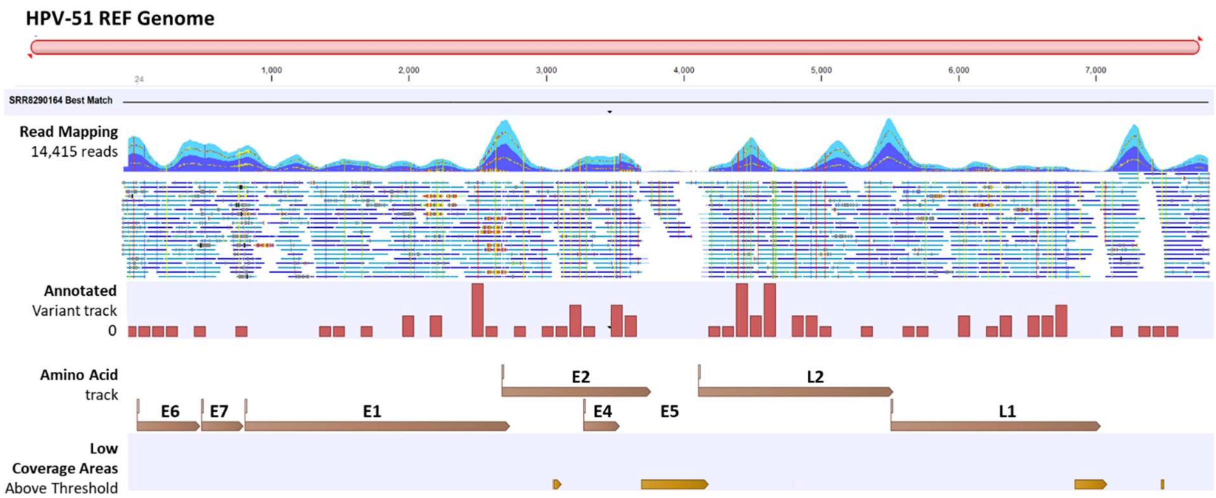

H S08 HSIL

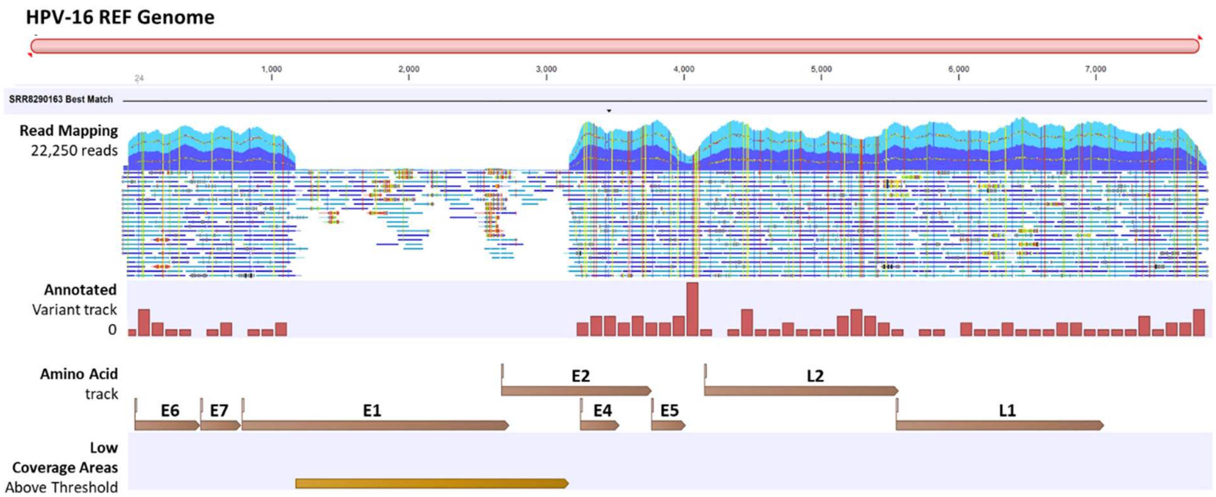

I S09 HSIL

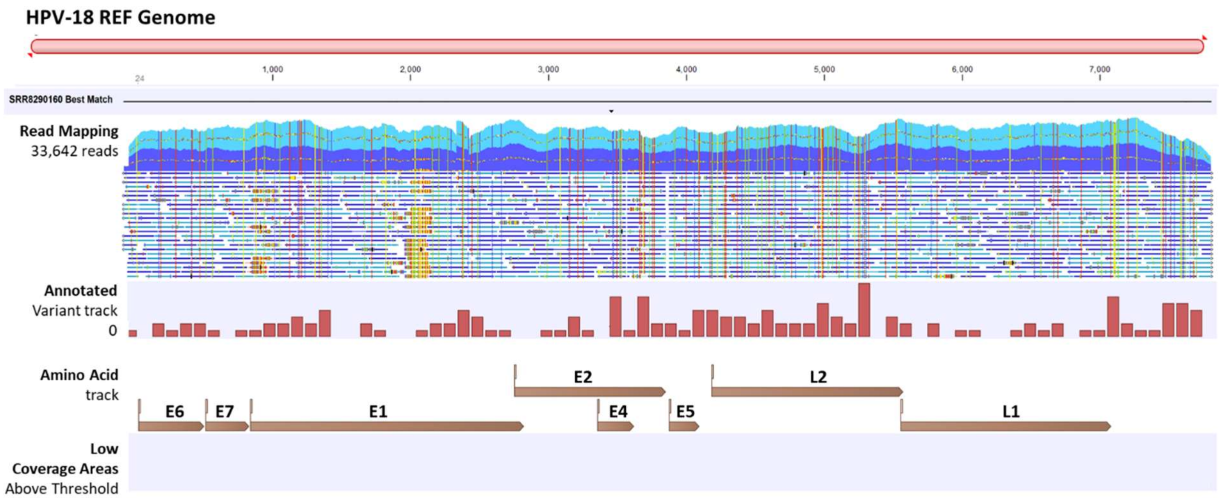

J S10 HSIL

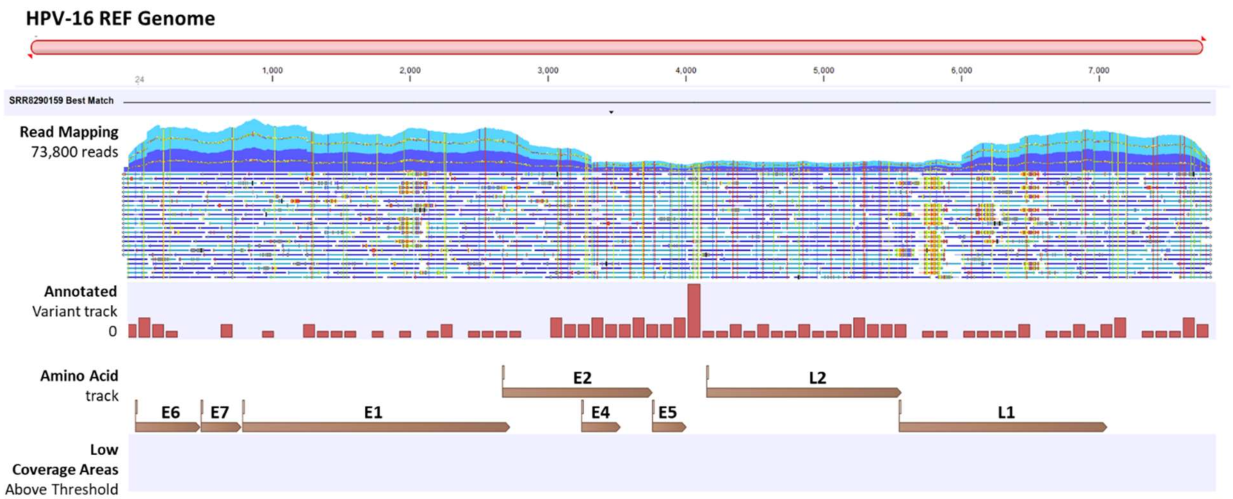

K S11 HSIL

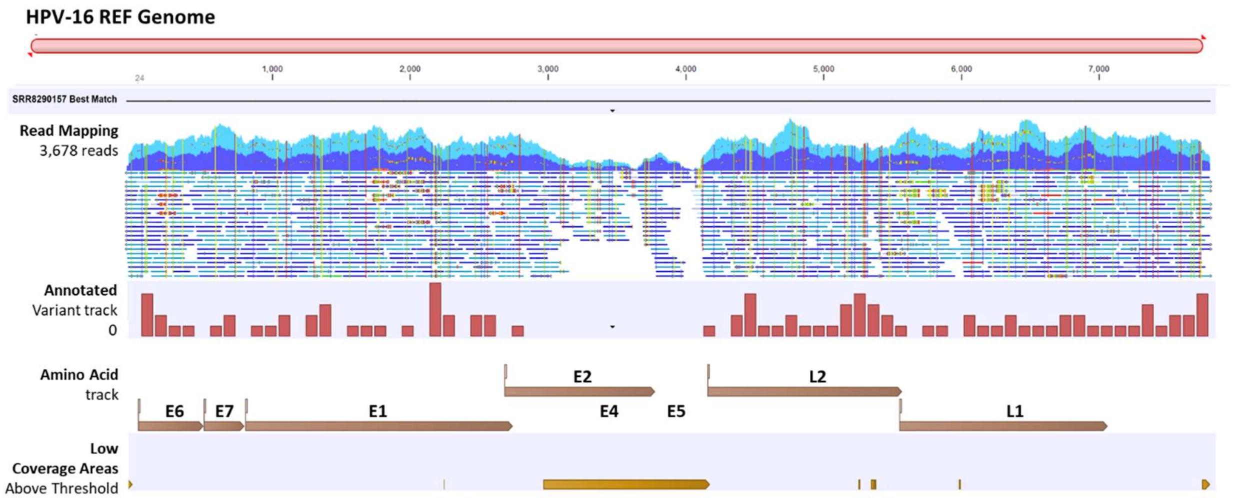

L S12 HSIL

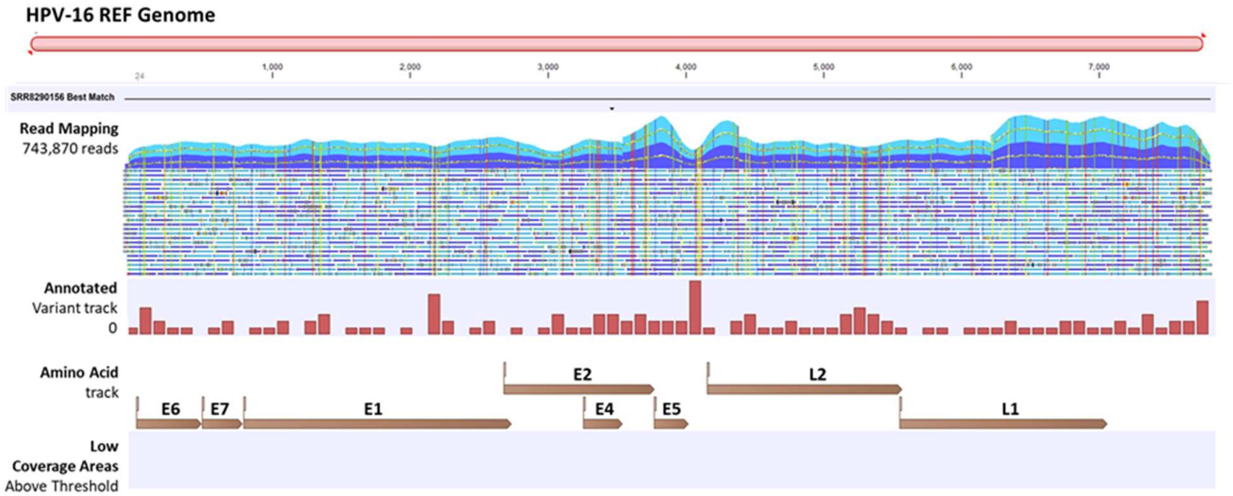

M S13 ASCH

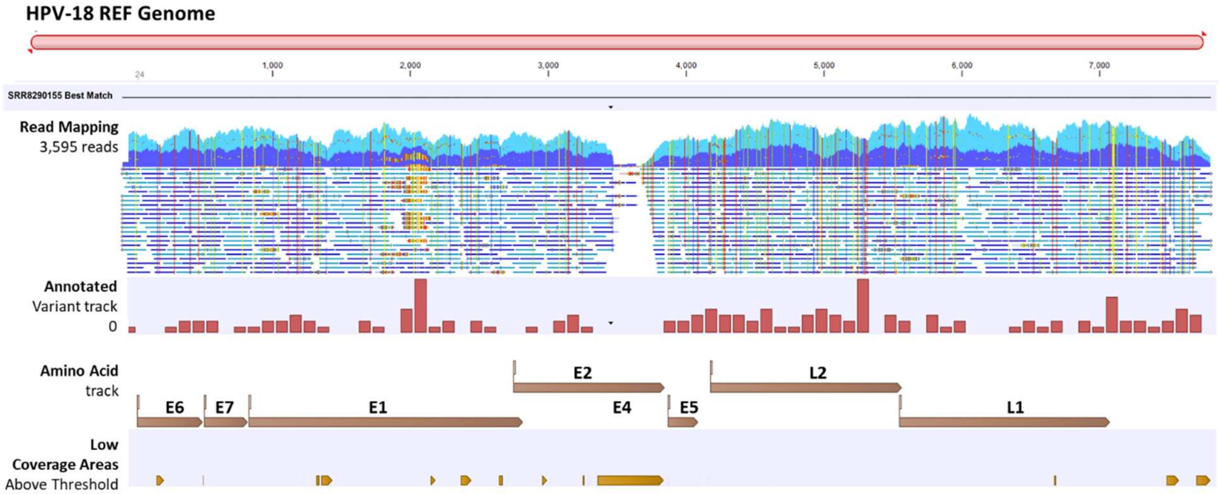

N S14 ASCH

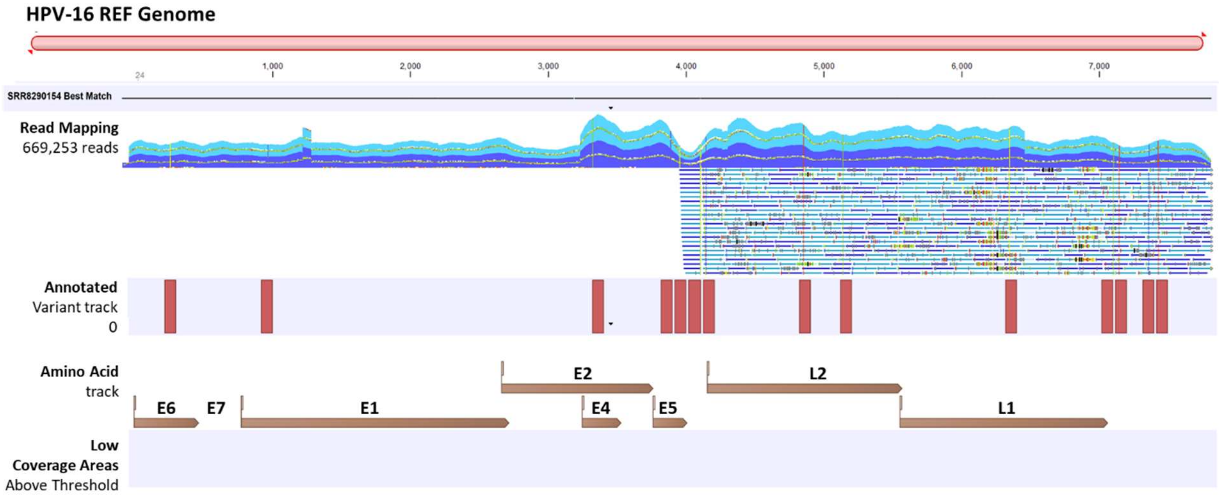

O S15 LSIL

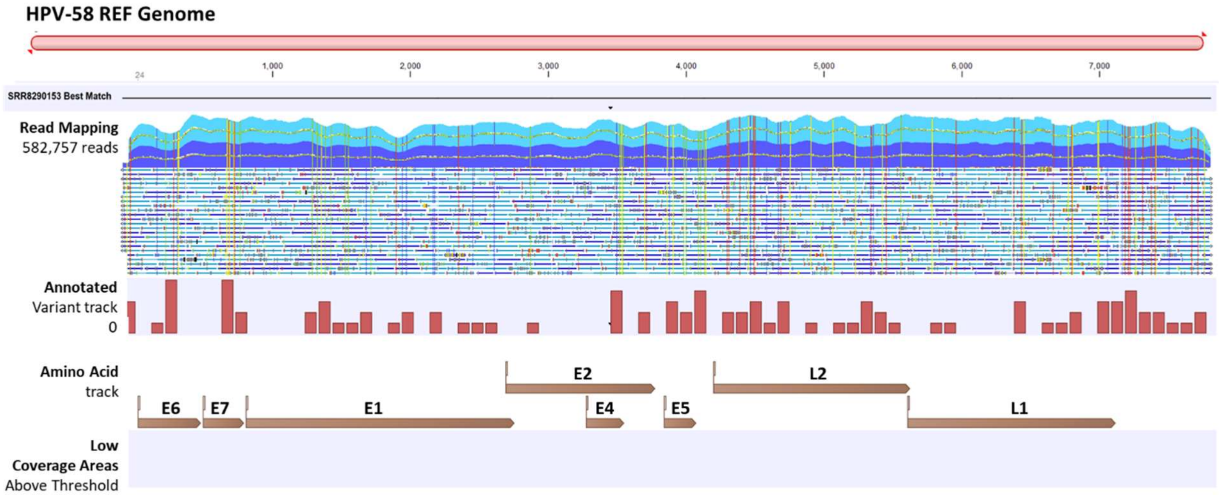

P S16 LSIL

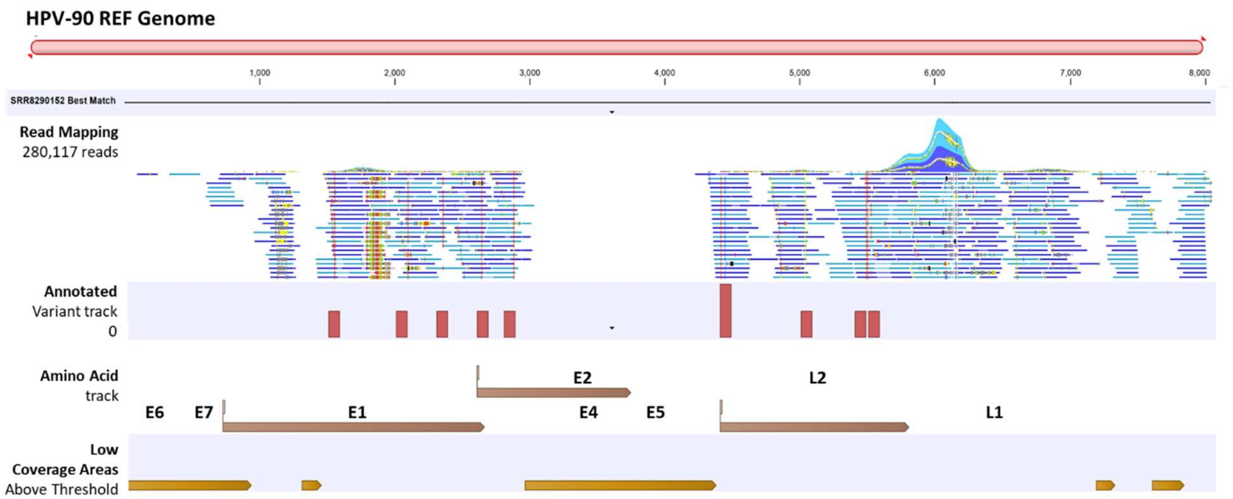

Q S17 LSIL

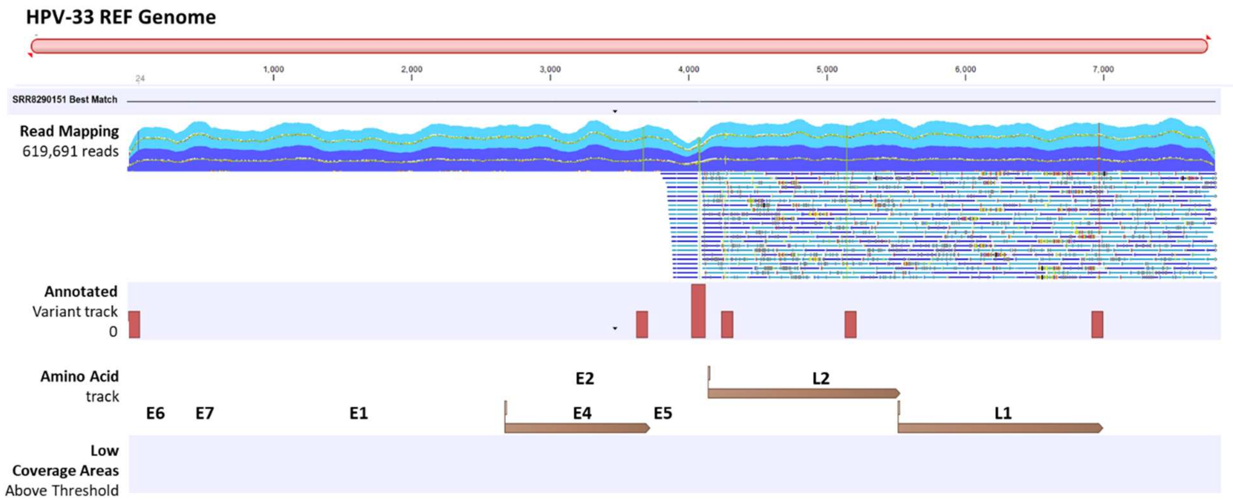

R S18 ASCUS

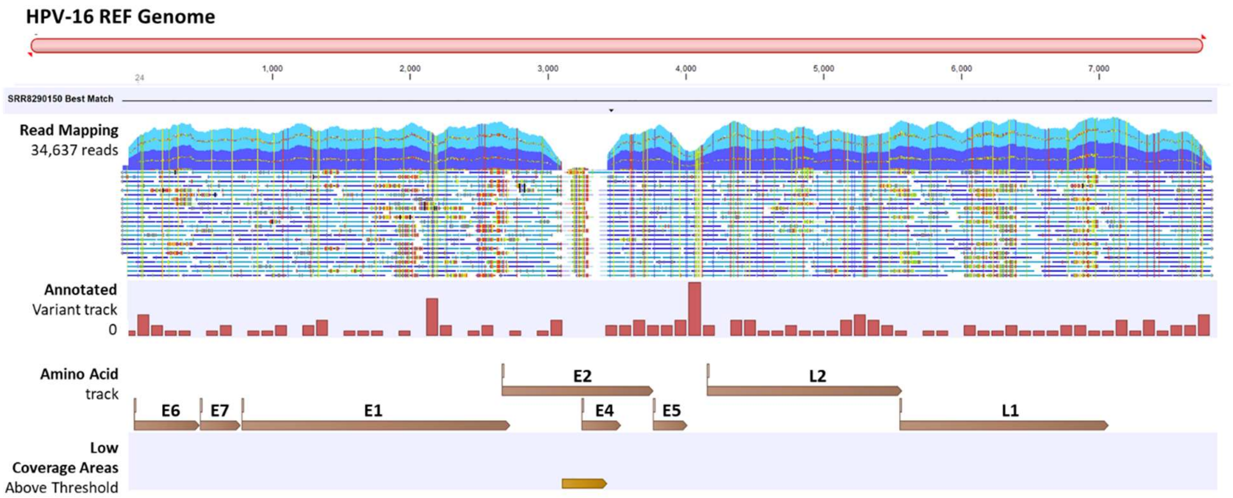

S S19 ASCUS

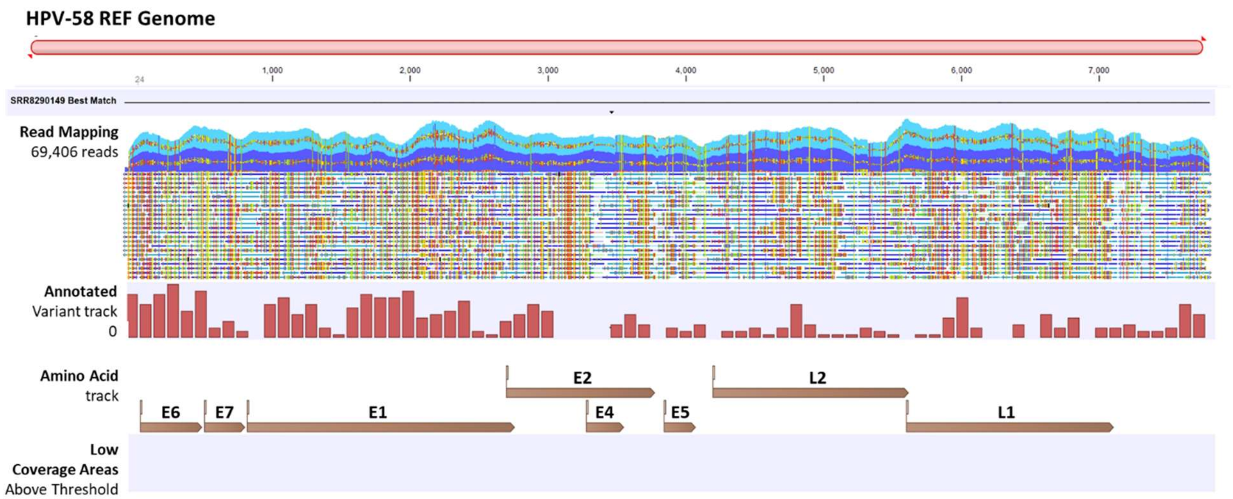

T S20 ASCUS

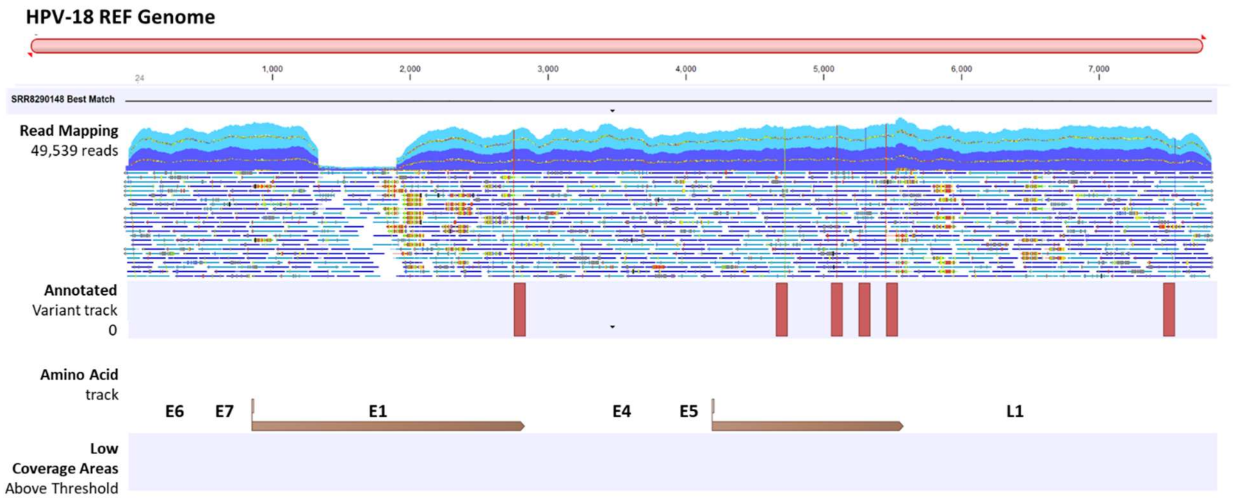

U S21 ASCUS

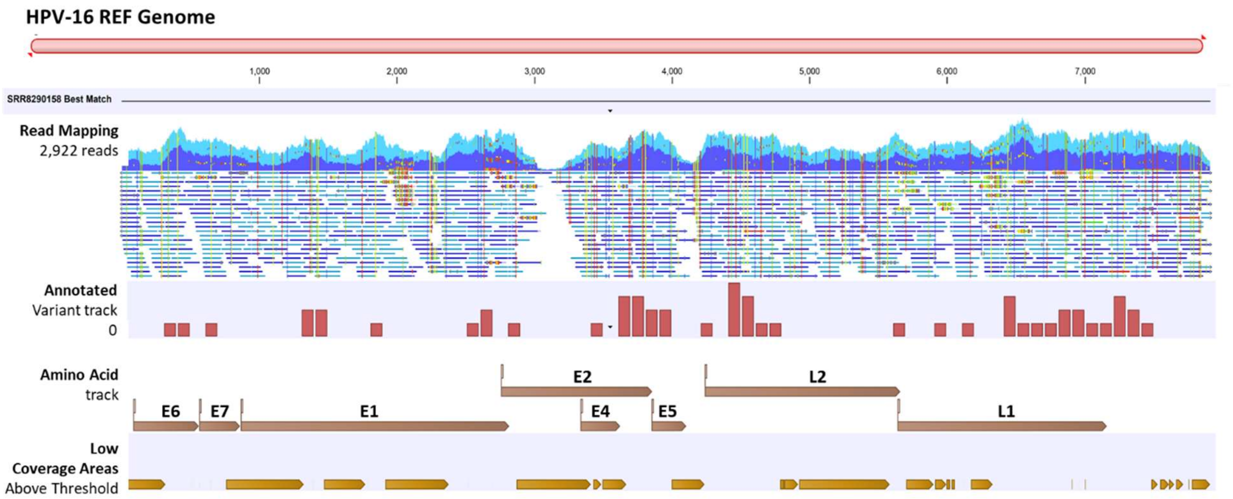

Figure S1. Viral Hybrid Capture (VHC) track list of the dominant HPV genotype identified by read mapping **(A-U)** Samples S01 to S21.
