# Supplementary material for: HPV Integration Site Mapping: A Rapid Method of Viral Integration Site (VIS) Analysis and Visualization Using Automated Workflows in CLC Microbial Genomics
Source: Int J Mol Sci. 2022 Jul 23;23(15):8132. doi: 10.3390/ijms23158132 (PMC9331699; doi:10.3390/ijms23158132)
Supplement: Supplementary file 1 [file ijms-23-08132-s001.zip › TABLE S1 HPV TAXON PROFILING.pdf]

Table S1 Taxonomic profiling results

Name	Taxonomy[1]	Combined Abundance	Min	Max	Mean	Median	Std
HPV16REF	Virus_dsDNA; Papillomaviridae; Alpha; Alpha 9; HPV16	15,286,336	0	10,588,208	727,921	5576	2,316,918
HPV18REF	Virus_dsDNA; Papillomaviridae; Alpha; Alpha 7; HPV18	243,834	0	117,956	11,611	0	30,489
HPV56REF	Virus_dsDNA; Papillomaviridae; Alpha; Alpha 6; HPV56	1,582	0	578	75	0	165
HPV33REF	Virus_dsDNA; Papillomaviridae; Alpha; Alpha 9; HPV33	2,659,324	0	2,613,114	126,634	0	569,746
HPV58REF	Virus_dsDNA; Papillomaviridae; Alpha; Alpha 9; HPV58	4,166,314	0	4,084,638	198,396	0	890,615
HPV51REF	Virus_dsDNA; Papillomaviridae; Alpha; Alpha 5; HPV51	167,204	0	151,076	7,962	0	32,979
HPV59REF	Virus_dsDNA; Papillomaviridae; Alpha; Alpha 7; HPV59	310	0	310	15	0	68
HPV90REF	Virus_dsDNA; Papillomaviridae; Alpha; Alpha 14; HPV90	2,232,342	0	2,227,900	106,302	0	486,120
HPV34REF	Virus_dsDNA; Papillomaviridae; Alpha; Alpha 11; HPV34	1,593,312	0	1,590,938	75,872	0	347,146
HPV82REF	Virus_dsDNA; Papillomaviridae; Alpha; Alpha 5; HPV82	106	0	106	5	0	23
HPV44REF	Virus_dsDNA; Papillomaviridae; Alpha; Alpha 10; HPV44	134,242	0	131,570	6,392	0	28,688
HPV68REF	Virus_dsDNA; Papillomaviridae; Alpha; Alpha 7; HPV68	5,382	0	5,382	256	0	1,174

REF, reference genome.

[1]An in-depth explanation of Taxonomic Profiling metrics (column headers) is provided in the CLC Microbial Genomics Module manual on-line [28].

[2]For samples S16 (SRR8290152) and S19 (SRR829149), the dominant genotypes were HPV-90 and HPV-58, while the integrated types were HPV -90 and -51, and HPV-18, respectively.

Table S1 Taxonomic profiling results

Name	SRR8290166 (trimmed pairs) Abundance	Coverage (SRR8290166 (trimmed pairs))	SRR8290165 (trimmed pairs) Abundance	Coverage (SRR8290165 (trimmed pairs))	SRR8290168 (trimmed pairs) Abundance	Coverage (SRR8290168 (trimmed pairs))	SRR8290167 (trimmed pairs) Abundance	Coverage (SRR8290167 (trimmed pairs))
HPV16REF	187706	3532.182393	778	14.213635	162586	3010.893246	423304	7591.024791
HPV18REF	0	0	13444	244.476645	0	0	0	0
HPV56REF	0	0	176	3.157935	0	0	0	0
HPV33REF	0	0	0	0	1202	22.123024	0	0
HPV58REF	0	0	0	0	0	0	0	0
HPV51REF	0	0	0	0	0	0	0	0
HPV59REF	0	0	0	0	0	0	0	0
HPV90REF	0	0	0	0	0	0	0	0
HPV34REF	0	0	0	0	0	0	0	0
HPV82REF	0	0	0	0	0	0	0	0
HPV44REF	0	0	0	0	0	0	0	0
HPV68REF	0	0	0	0	0	0	0	0

Table S1 Taxonomic profiling results

Name	SRR8290162 (trimmed pairs) Abundance	Coverage (SRR8290162 (trimmed pairs))	SRR8290161 (trimmed pairs) Abundance	Coverage (SRR8290161 (trimmed pairs))	SRR8290164 (trimmed pairs) Abundance	Coverage (SRR8290164 (trimmed pairs))	SRR8290163 (trimmed pairs) Abundance	Coverage (SRR8290163 (trimmed pairs))
HPV16REF	1781450	33030.74538	146288	2752.868075	490	9.110296	33998	621.068808
HPV18REF	0	0	0	0	0	0	0	0
HPV56REF	0	0	0	0	0	0	482	8.7058
HPV33REF	0	0	0	0	8012	148.693134	0	0
HPV58REF	0	0	1538	28.465235	0	0	402	7.384586
HPV51REF	0	0	0	0	16128	304.873207	0	0
HPV59REF	0	0	0	0	310	5.700608	0	0
HPV90REF	0	0	0	0	0	0	0	0
HPV34REF	0	0	0	0	0	0	0	0
HPV82REF	0	0	0	0	0	0	0	0
HPV44REF	0	0	0	0	0	0	0	0
HPV68REF	0	0	0	0	0	0	0	0

Table S1 Taxonomic profiling results

Name	SRR8290160 (trimmed pairs) Abundance	Coverage (SRR8290160 (trimmed pairs))	SRR8290159 (trimmed pairs) Abundance	Coverage (SRR8290159 (trimmed pairs))	SRR8290157 (trimmed pairs) Abundance	Coverage (SRR8290157 (trimmed pairs))	SRR8290156 (trimmed pairs) Abundance	Coverage (SRR8290156 (trimmed pairs))
HPV16REF	4932	90.701872	203268	3842.209714	5576	102.017455	10588208	194590.8053
HPV18REF	81774	1550.895634	0	0	0	0	0	0
HPV56REF	0	0	0	0	578	10.205991	0	0
HPV33REF	0	0	0	0	0	0	0	0
HPV58REF	0	0	0	0	378	6.783742	0	0
HPV51REF	0	0	0	0	0	0	0	0
HPV59REF	0	0	0	0	0	0	0	0
HPV90REF	0	0	3324	61.721648	0	0	0	0
HPV34REF	0	0	2374	45.914153	0	0	0	0
HPV82REF	0	0	0	0	106	1.918043	0	0
HPV44REF	0	0	0	0	0	0	0	0
HPV68REF	0	0	0	0	0	0	0	0

Table S1 Taxonomic profiling results

Name	SRR8290155 Abundance	Coverage (SRR8290155)	SRR8290154 Abundance	Coverage (SRR8290154)	SRR8290153 Abundance	Coverage (SRR8290153)	SRR8290152[2] Abundance	Coverage (SRR8290152)
HPV16REF	750	13.760815	1702732	31431.93284	0	0	0	0
HPV18REF	6062	112.191676	0	0	0	0	0	0
HPV56REF	114	2.038241	0	0	0	0	0	0
HPV33REF	264	4.864585	10386	192.169048	0	0	0	0
HPV58REF	164	2.971115	0	0	4084638	75594.38727	0	0
HPV51REF	0	0	0	0	0	0	151076	2890.558017
HPV59REF	0	0	0	0	0	0	0	0
HPV90REF	0	0	0	0	0	0	2227900	41371.03411
HPV34REF	0	0	0	0	0	0	1590938	30750.5147
HPV82REF	0	0	0	0	0	0	0	0
HPV44REF	0	0	0	0	131570	2461.248691	0	0
HPV68REF	0	0	0	0	0	0	0	0

Table S1 Taxonomic profiling results

Name	SRR8290151 Abundance	Coverage (SRR8290151)	SRR8290150 Abundance	Coverage (SRR8290150)	SRR8290149[2] Abundance	Coverage (SRR8290149)	SRR8290148 Abundance	Coverage (SRR8290148)
HPV16REF	0	0	40148	747.163547	0	0	0	0
HPV18REF	0	0	0	0	24598	451.483263	117956	2216.956217
HPV56REF	0	0	0	0	0	0	0	0
HPV33REF	2613114	48103.94551	5428	100.327601	19890	366.832975	1028	18.937792
HPV58REF	0	0	0	0	78782	1465.896345	0	0
HPV51REF	0	0	0	0	0	0	0	0
HPV59REF	0	0	0	0	0	0	0	0
HPV90REF	0	0	0	0	0	0	1118	20.729242
HPV34REF	0	0	0	0	0	0	0	0
HPV82REF	0	0	0	0	0	0	0	0
HPV44REF	0	0	0	0	2672	50.149368	0	0
HPV68REF	0	0	0	0	5382	100.022884	0	0

Table S1 Taxonomic profiling results

Name	SRR8290158 (trimmed pairs) Abundance	Coverage (SRR8290158 (trimmed pairs))
HPV16REF	4122	74.447761
HPV18REF	0	0
HPV56REF	232	4.152326
HPV33REF	0	0
HPV58REF	412	7.619121
HPV51REF	0	0
HPV59REF	0	0
HPV90REF	0	0
HPV34REF	0	0
HPV82REF	0	0
HPV44REF	0	0
HPV68REF	0	0
