# Supplementary material for: HPV Integration Site Mapping: A Rapid Method of Viral Integration Site (VIS) Analysis and Visualization Using Automated Workflows in CLC Microbial Genomics
Source: Int J Mol Sci. 2022 Jul 23;23(15):8132. doi: 10.3390/ijms23158132 (PMC9331699; doi:10.3390/ijms23158132)
Supplement: Supplementary file 1 [file ijms-23-08132-s001.zip › TABLE S2 BLAST HPV SUBLINEAGES.pdf]

Table S2 BLAST identification of HPV sublineages

Query[1]	Dominant, Integrated genotype	No. of HSPs	Lowest E-value	Accession (E-value)	Description (E-value)
SRR8290148 Read Mapping HPV18REF consensus	Int, D=I	994	0	HPV18,_A1REF_AY262282	HPV 18 complete sequence.
SRR8290149 (Viral mappings) HPV18REF consensus	Int, D≠I	978	0	HPV18,_B3REF_EF202152	HPV 18 isolate Qv17199, complete genome.
SRR8290150 Read Mapping HPV16REF consensus	Int, D=I	1003	0	HPV16,_C1REF,_AF2_AF472509	HPV 16 variant (African type 2), complete genome.
SRR8290151 Read Mapping HPV33REF consensus	Int, D=I	1056	0	HPV33,_A1REF_PPH33CG	HPV 33, complete genome.
SRR8290152 (Viral mappings) HPV51REF consensus	Int, D≠I	789	0	HPV51,B1REF_KF436883	HPV 51 isolate Qv10565, complete genome.
SRR8290153 Read Mapping HPV58REF consensus	Dom	1036	0	HPV58,_D2REF_HQ537770	HPV 58 isolate Rw697, complete genome.
SRR8290154 Read Mapping HPV16REF consensus	Int, D=I	895	0	HPV16,_A3REF_EU_HQ644236	HPV 16 isolate AS411, complete genome.
SRR8290155 Read Mapping HPV18REF consensus	Dom	924	0	HPV18,_B2REF_KC470225	HPV 18 isolate BF172, complete genome.
SRR8290156 Read Mapping HPV16REF consensus	Int, D=I	886	0	HPV16,_C1REF,_AF2_AF472509	HPV 16 variant (African type 2), complete genome.
SRR8290157 Read Mapping HPV16REF consensus	Dom	822	0	HPV16,_C1REF,_AF2_AF472509	HPV 16 variant (African type 2), complete genome.
SRR8290158 Read Mapping HPV16REF consensus	Dom	1262	0	HPV16,_C1REF,_AF2_AF472509	HPV 16 variant (African type 2), complete genome.
SRR8290159 Read Mapping HPV16REF consensus	Int, D=I	913	0	HPV16,_B1REF_AF1_AF536180	HPV 16 isolate African-1 type, complete genome.
SRR8290160 Read Mapping HPV18REF consensus	Int, D=I	979	0	HPV18,_B2REF_KC470225	HPV 18 isolate BF172, complete genome.
SRR8290161 Read Mapping HPV16REF consensus	Int, D=I	892	0	HPV16,_A3REF_EU_HQ644236	HPV 16 isolate AS411, complete genome.
SRR8290162 Read Mapping HPV16REF consensus	Int, D=I	885	0	HPV16,_C1REF,_AF2_AF472509	HPV 16 variant (African type 2), complete genome.
SRR8290163 Read Mapping HPV16REF consensus	Int, D=I	962	0	HPV16,_C1REF,_AF2_AF472509	HPV 16 variant (African type 2), complete genome.
SRR8290164 Read Mapping HPV51REF consensus	Dom	848	0	HPV51,B1REF_KF436883	HPV 51 isolate Qv10565, complete genome.
SRR8290165 Read Mapping HPV18REF consensus	Int, D=I	709	0	HPV18,_C1REF_KC470229	HPV 18 isolate Qv39775, complete genome.
SRR8290166 Read Mapping HPV16REF consensus	Int, D=I	564	0	HPV16,_B2REF_AF1_HQ644298	HPV 16 isolate Z109, complete genome.
SRR8290167 Read Mapping HPV16REF consensus	Dom	905	0	HPV16,_B1REF_AF1_AF536180	HPV 16 isolate African-1 type, complete genome.
SRR8290168 Read Mapping HPV16REF consensus	Int, D=I	1155	0	HPV16,_C1REF,_AF2_AF472509	HPV 16 variant (African type 2), complete genome.

Dom, dominant HPV genotype; D = I, dominant and integrated HPV types are identical; D /= I, dominant and integrated HPV types are nonidentical; Int, integrated HPV genotype; REF, reference

[1]Query sequences and BLAST results. BLAST statistics nomenclature and definitions from left to right of table columns [39]: HSP (n), Number of high scoring pairs from the source database; Lowest E-val, Lowest Expect value; Accession (E-val), Accession name of the matched database sequence with the lowest E-value; HPV Type, HPV genotype (numeral), variant and sub-lineage (alphanumeric) of the Accession sequence; Hit, Name of the sequence found in the BLAST search with the max score; Total score, Total alignment score for all HSPs; Max score, Maximum (best) score or highest alignment score of all HSPs; Min E-value, Minimum (best) e-value of all HSPs; Max bit score, Maximum (best) bit score of all HSPs; Max id, Maximum number of identical residues in the query and Hit sequence; Max %id, Percentage of maximum identical residues in the query and Hit sequence; Max pos, Maximum number of similar but not necessarily identical residues in the query and Hit sequence; Max %pos, Percentage of maximum similar but not necessarily identical residues in the query and Hit sequence.

Table S2 BLAST identification of HPV sublineages

Query	Greatest identity %	Accession (identity %)	Description (identity %)
SRR8290148 Read Mapping HPV18REF consensus	100	HPV30,_A1REF_X74474	HPV 30 genomic DNA.
SRR8290149 (Viral mappings) HPV18REF consensus	100	HPV30,_A1REF_X74474	HPV 30 genomic DNA.
SRR8290150 Read Mapping HPV16REF consensus	100	HPV33,_A3REF_EU918766	HPV 33 isolate LZcc12-33, complete genome.
SRR8290151 Read Mapping HPV33REF consensus	100	HPV67,_A1REF_D21208	HPV 67 complete genome.
SRR8290152 (Viral mappings) HPV51REF consensus	100	HPV35,_A1REF_X74477	HPV 35H genomic DNA.
SRR8290153 Read Mapping HPV58REF consensus	100	HPV35,_A2REF_HQ537727	HPV 35 isolate Rw128, complete genome.
SRR8290154 Read Mapping HPV16REF consensus	100	HPV52,_C2REF_HQ537746	HPV 52 isolate Qv00615, complete genome.
SRR8290155 Read Mapping HPV18REF consensus	100	HPV30,_A1REF_X74474	HPV 30 genomic DNA.
SRR8290156 Read Mapping HPV16REF consensus	100	HPV54,_C1REF_KF436894	HPV 54 isolate Qv18028, complete genome.
SRR8290157 Read Mapping HPV16REF consensus	100	HPV54,_C1REF_KF436894	HPV 54 isolate Qv18028, complete genome.
SRR8290158 Read Mapping HPV16REF consensus	100	HPV54,_C1REF_KF436894	HPV 54 isolate Qv18028, complete genome.
SRR8290159 Read Mapping HPV16REF consensus	100	HPV54,_C1REF_KF436894	HPV 54 isolate Qv18028, complete genome.
SRR8290160 Read Mapping HPV18REF consensus	100	HPV30,_A1REF_X74474	HPV 30 genomic DNA.
SRR8290161 Read Mapping HPV16REF consensus	100	HPV54,_C1REF_KF436894	HPV 54 isolate Qv18028, complete genome.
SRR8290162 Read Mapping HPV16REF consensus	100	HPV54,_C1REF_KF436894	HPV 54 isolate Qv18028, complete genome.
SRR8290163 Read Mapping HPV16REF consensus	100	HPV16,_C1REF,_AF2_AF472509	HPV 16 variant (African type 2), complete genome.
SRR8290164 Read Mapping HPV51REF consensus	100	HPV30,_A3REF_KF436844	HPV 30 isolate Rw060, complete genome.
SRR8290165 Read Mapping HPV18REF consensus	100	HPV54,_B1REF_AF436129	HPV 54 subtype AE9, complete genome.
SRR8290166 Read Mapping HPV16REF consensus	100	HPV54,_C1REF_KF436894	HPV 54 isolate Qv18028, complete genome.
SRR8290167 Read Mapping HPV16REF consensus	100	HPV54,_C1REF_KF436894	HPV 54 isolate Qv18028, complete genome.
SRR8290168 Read Mapping HPV16REF consensus	100	HPV16,_A3REF_EU_HQ644236	HPV 16 isolate AS411, complete genome.

Table S2 BLAST identification of HPV sublineages

Query	Greatest positive %	Accession (positive %)	Description (positive %)
SRR8290148 Read Mapping HPV18REF consensus	100	HPV30,_A1REF_X74474	HPV 30 genomic DNA.
SRR8290149 (Viral mappings) HPV18REF consensus	100	HPV30,_A1REF_X74474	HPV 30 genomic DNA.
SRR8290150 Read Mapping HPV16REF consensus	100	HPV33,_A3REF_EU918766	HPV 33 isolate LZcc12-33, complete genome.
SRR8290151 Read Mapping HPV33REF consensus	100	HPV67,_A1REF_D21208	HPV 67 complete genome.
SRR8290152 (Viral mappings) HPV51REF consensus	100	HPV35,_A1REF_X74477	HPV 35H genomic DNA.
SRR8290153 Read Mapping HPV58REF consensus	100	HPV35,_A2REF_HQ537727	HPV 35 isolate Rw128, complete genome.
SRR8290154 Read Mapping HPV16REF consensus	100	HPV52,_C2REF_HQ537746	HPV 52 isolate Qv00615, complete genome.
SRR8290155 Read Mapping HPV18REF consensus	100	HPV30,_A1REF_X74474	HPV 30 genomic DNA.
SRR8290156 Read Mapping HPV16REF consensus	100	HPV54,_C1REF_KF436894	HPV 54 isolate Qv18028, complete genome.
SRR8290157 Read Mapping HPV16REF consensus	100	HPV54,_C1REF_KF436894	HPV 54 isolate Qv18028, complete genome.
SRR8290158 Read Mapping HPV16REF consensus	100	HPV54,_C1REF_KF436894	HPV 54 isolate Qv18028, complete genome.
SRR8290159 Read Mapping HPV16REF consensus	100	HPV54,_C1REF_KF436894	HPV 54 isolate Qv18028, complete genome.
SRR8290160 Read Mapping HPV18REF consensus	100	HPV30,_A1REF_X74474	HPV 30 genomic DNA.
SRR8290161 Read Mapping HPV16REF consensus	100	HPV54,_C1REF_KF436894	HPV 54 isolate Qv18028, complete genome.
SRR8290162 Read Mapping HPV16REF consensus	100	HPV54,_C1REF_KF436894	HPV 54 isolate Qv18028, complete genome.
SRR8290163 Read Mapping HPV16REF consensus	100	HPV16,_C1REF,_AF2_AF472509	HPV 16 variant (African type 2), complete genome.
SRR8290164 Read Mapping HPV51REF consensus	100	HPV30,_A3REF_KF436844	HPV 30 isolate Rw060, complete genome.
SRR8290165 Read Mapping HPV18REF consensus	100	HPV54,_B1REF_AF436129	HPV 54 subtype AE9, complete genome.
SRR8290166 Read Mapping HPV16REF consensus	100	HPV54,_C1REF_KF436894	HPV 54 isolate Qv18028, complete genome.
SRR8290167 Read Mapping HPV16REF consensus	100	HPV54,_C1REF_KF436894	HPV 54 isolate Qv18028, complete genome.
SRR8290168 Read Mapping HPV16REF consensus	100	HPV16,_A3REF_EU_HQ644236	HPV 16 isolate AS411, complete genome.

Table S2 BLAST identification of HPV sublineages

Query	Greatest HSP length	Accession (HSP length)	Description (HSP length)
SRR8290148 Read Mapping HPV18REF consensus	7857	HPV18,_A1REF_AY262282	HPV 18 complete sequence.
SRR8290149 (Viral mappings) HPV18REF consensus	7857	HPV18,_A2REF_EF202146	HPV 18 isolate Qv16306, complete genome.
SRR8290150 Read Mapping HPV16REF consensus	4389	HPV16,_B2REF_AF1_HQ644298	HPV 16 isolate Z109, complete genome.
SRR8290151 Read Mapping HPV33REF consensus	7909	HPV33,_A1REF_PPH33CG	HPV 33, complete genome.
SRR8290152 (Viral mappings) HPV51REF consensus	7901	HPV82,_C5REF_KF436803	HPV 82 isolate Qv28248, complete genome.
SRR8290153 Read Mapping HPV58REF consensus	7836	HPV58,_A3REF_HQ537758	HPV 58 isolate Qv00961, complete genome.
SRR8290154 Read Mapping HPV16REF consensus	7909	HPV16,_B2REF_AF1_HQ644298	HPV 16 isolate Z109, complete genome.
SRR8290155 Read Mapping HPV18REF consensus	3961	HPV18,_A3REF_EF202147	HPV 18 isolate Qv15586, complete genome.
SRR8290156 Read Mapping HPV16REF consensus	7909	HPV16,_B2REF_AF1_HQ644298	HPV 16 isolate Z109, complete genome.
SRR8290157 Read Mapping HPV16REF consensus	3705	HPV16,_C1REF,_AF2_AF472509	HPV 16 variant (African type 2), complete genome.
SRR8290158 Read Mapping HPV16REF consensus	3664	HPV16,_C1REF,_AF2_AF472509	HPV 16 variant (African type 2), complete genome.
SRR8290159 Read Mapping HPV16REF consensus	7909	HPV16,_B2REF_AF1_HQ644298	HPV 16 isolate Z109, complete genome.
SRR8290160 Read Mapping HPV18REF consensus	7856	HPV18,_A3REF_EF202147	HPV 18 isolate Qv15586, complete genome.
SRR8290161 Read Mapping HPV16REF consensus	7909	HPV16,_B2REF_AF1_HQ644298	HPV 16 isolate Z109, complete genome.
SRR8290162 Read Mapping HPV16REF consensus	7909	HPV16,_B2REF_AF1_HQ644298	HPV 16 isolate Z109, complete genome.
SRR8290163 Read Mapping HPV16REF consensus	4658	HPV16,_B2REF_AF1_HQ644298	HPV 16 isolate Z109, complete genome.
SRR8290164 Read Mapping HPV51REF consensus	3781	HPV26,A1REF_X74472	HPV 26 genomic DNA.
SRR8290165 Read Mapping HPV18REF consensus	1413	HPV18,_C1REF_KC470229	HPV 18 isolate Qv39775, complete genome.
SRR8290166 Read Mapping HPV16REF consensus	3229	HPV16,_B2REF_AF1_HQ644298	HPV 16 isolate Z109, complete genome.
SRR8290167 Read Mapping HPV16REF consensus	7909	HPV16,_B2REF_AF1_HQ644298	HPV 16 isolate Z109, complete genome.
SRR8290168 Read Mapping HPV16REF consensus	3726	HPV16,_D1REF,_NA_HQ644257	HPV 16 isolate Qv00512, complete genome.

Table S2 BLAST identification of HPV sublineages

Query	Greatest bit score	Accession (bit score)	Description (bit score)
SRR8290148 Read Mapping HPV18REF consensus	14143.3	HPV18,_A1REF_AY262282	HPV 18 complete sequence.
SRR8290149 (Viral mappings) HPV18REF consensus	13883.6	HPV18,_B3REF_EF202152	HPV 18 isolate Qv17199, complete genome.
SRR8290150 Read Mapping HPV16REF consensus	7824.3	HPV16,_C1REF,_AF2_AF472509	HPV 16 variant (African type 2), complete genome.
SRR8290151 Read Mapping HPV33REF consensus	14236.2	HPV33,_A1REF_PPH33CG	HPV 33, complete genome.
SRR8290152 (Viral mappings) HPV51REF consensus	14045	HPV51,B1REF_KF436883	HPV 51 isolate Qv10565, complete genome.
SRR8290153 Read Mapping HPV58REF consensus	14012.6	HPV58,_D2REF_HQ537770	HPV 58 isolate Rw697, complete genome.
SRR8290154 Read Mapping HPV16REF consensus	14139.7	HPV16,_A3REF_EU_HQ644236	HPV 16 isolate AS411, complete genome.
SRR8290155 Read Mapping HPV18REF consensus	7059.67	HPV18,_B2REF_KC470225	HPV 18 isolate BF172, complete genome.
SRR8290156 Read Mapping HPV16REF consensus	14183	HPV16,_C1REF,_AF2_AF472509	HPV 16 variant (African type 2), complete genome.
SRR8290157 Read Mapping HPV16REF consensus	6669.24	HPV16,_C1REF,_AF2_AF472509	HPV 16 variant (African type 2), complete genome.
SRR8290158 Read Mapping HPV16REF consensus	6409.56	HPV16,_C1REF,_AF2_AF472509	HPV 16 variant (African type 2), complete genome.
SRR8290159 Read Mapping HPV16REF consensus	14133.4	HPV16,_B1REF_AF1_AF536180	HPV 16 isolate African-1 type, complete genome.
SRR8290160 Read Mapping HPV18REF consensus	14023.4	HPV18,_B2REF_KC470225	HPV 18 isolate BF172, complete genome.
SRR8290161 Read Mapping HPV16REF consensus	14146.9	HPV16,_A3REF_EU_HQ644236	HPV 16 isolate AS411, complete genome.
SRR8290162 Read Mapping HPV16REF consensus	14237.1	HPV16,_C1REF,_AF2_AF472509	HPV 16 variant (African type 2), complete genome.
SRR8290163 Read Mapping HPV16REF consensus	8378.84	HPV16,_C1REF,_AF2_AF472509	HPV 16 variant (African type 2), complete genome.
SRR8290164 Read Mapping HPV51REF consensus	6575.47	HPV51,B1REF_KF436883	HPV 51 isolate Qv10565, complete genome.
SRR8290165 Read Mapping HPV18REF consensus	2544.94	HPV18,_C1REF_KC470229	HPV 18 isolate Qv39775, complete genome.
SRR8290166 Read Mapping HPV16REF consensus	5748.62	HPV16,_B2REF_AF1_HQ644298	HPV 16 isolate Z109, complete genome.
SRR8290167 Read Mapping HPV16REF consensus	14139.7	HPV16,_B1REF_AF1_AF536180	HPV 16 isolate African-1 type, complete genome.
SRR8290168 Read Mapping HPV16REF consensus	6533.99	HPV16,_C1REF,_AF2_AF472509	HPV 16 variant (African type 2), complete genome.
