# Supplementary material for: HPV Integration Site Mapping: A Rapid Method of Viral Integration Site (VIS) Analysis and Visualization Using Automated Workflows in CLC Microbial Genomics
Source: Int J Mol Sci. 2022 Jul 23;23(15):8132. doi: 10.3390/ijms23158132 (PMC9331699; doi:10.3390/ijms23158132)
Supplement: Supplementary file 1 [file ijms-23-08132-s001.zip › TABLE S3 VIRAL INT SITES REPORTS.pdf]

Table S3. Viral integration sites summary reports

S01_SRR8290166 (Report)

1. Identify Viral Integration Sites summary

Input reads	290,140
Host reads	84,430
Virus reads	192,175
Unmapped reads	13,535
Host reads (%)	29.10
Virus reads (%)	66.24
Unmapped reads (%)	4.66
Breakpoints identified	2
Viruses identified	1

2. Identify Viral Integration Sites virus content

Virus	Reads mapped
HPV16REF	97,580

3. Identify Viral Integration Sites breakpoint summary

Host chromosome	Host region	Virus	Virus region	Unaligned ends host
11	complement (4119916..4119917)	HPV16REF	complement(5538.. 5539)	628
11	4119907..4119908	HPV16REF	3229..3230	382

Host chromosome	Unaligned ends virus	Broken reads host	Disrupted genes	Nearby genes
11	1,504	628	RRM1	STIM1 (-26706), RRM1
11	388	236	RRM1	STIM1 (-26697), RRM1

1. Identify Viral Integration Sites summary

Input reads	36,804
Host reads	20,999
Virus reads	15,178
Unmapped reads	627
Host reads (%)	57.06
Virus reads (%)	41.24
Unmapped reads (%)	1.70
Breakpoints identified	2
Viruses identified	5

2. Identify Viral Integration Sites virus content

Virus	Reads mapped
HPV18REF	7,043
HPV16REF	414
HPV56REF	96
HPV54REF	84
HPV68REF	71

3. Identify Viral Integration Sites breakpoint summary

Host chromosome	Host region	Virus	Virus region	Unaligned ends host
13	73396391..73396392	HPV18REF	complement(6480..6481)	75
20	complement (54060461..54060462)	HPV18REF	1413..1414	54

Host chromosome	Unaligned ends virus	Broken reads host	Disrupted genes	Nearby genes
13	169	32		gene: ENSG00000279754 (+2639)
20	152	33	BCAS1	BCAS1, CYP24A1 (+92984)

1. Identify Viral Integration Sites summary

Input reads	186,028
Host reads	17,278
Virus reads	167,018
Unmapped reads	1,732
Host reads (%)	9.29
Virus reads (%)	89.78
Unmapped reads (%)	0.93
Breakpoints identified	2
Viruses identified	1

2. Identify Viral Integration Sites virus content

Virus	Reads mapped
HPV16REF	84,624

3. Identify Viral Integration Sites breakpoint summary

Host chromosome	Host region	Virus	Virus region	Unaligned ends host
8	complement (42807080..42807081)	HPV16REF	2496..2497	493
8	42807078..42807079	HPV16REF	complement(5459.. 5460)	503

Host chromosome	Unaligned ends virus	Broken reads host	Disrupted genes	Nearby genes
8	1,193	333		CHRNB3 (-69673), CHRNA6 (-10688), THAP1 (+29593), RNF170 (+42556), HOOK3 (+89865)
8	1,043	358		CHRNB3 (-69671), CHRNA6 (-10686), THAP1 (+29595), RNF170 (+42558), HOOK3 (+89867)

1. Identify Viral Integration Sites summary

Input reads	452,888
Host reads	20,181
Virus reads	430,670
Unmapped reads	2,037
Host reads (%)	4.46
Virus reads (%)	95.09
Unmapped reads (%)	0.45
Breakpoints identified	0
Viruses identified	1

2. Identify Viral Integration Sites virus content

Virus	Reads mapped
HPV16REF	217,149

3. Identify Viral Integration Sites breakpoint summary

1. Identify Viral Integration Sites summary

Input reads	1,898,638
Host reads	81,161
Virus reads	1,810,829
Unmapped reads	6,648
Host reads (%)	4.27
Virus reads (%)	95.38
Unmapped reads (%)	0.35
Breakpoints identified	5
Viruses identified	1

2. Identify Viral Integration Sites virus content

Virus	Reads mapped
HPV16REF	911,568

3. Identify Viral Integration Sites breakpoint summary

Host chromosome	Host region	Virus	Virus region	Unaligned ends host
2	15898662..15898663	HPV16REF	7162..7163	25
2	15938439..15938440	HPV16REF	2328..2329	60
3	complement (94619627..94619628)	HPV16REF	5583..5584	40
3	complement (94854453..94854454)	HPV16REF	1529..1530	33
14	complement (99354484..99354485)	HPV16REF	2771..2772	20

Host chromosome	Unaligned ends virus	Broken reads host	Disrupted genes	Nearby genes
2	N/A	8		MYCN (+41887)
2	N/A	44		MYCN (+2110)
3	N/A	8		
3	N/A	26		

S05_SRR8290162 (Report)

Host chromosome	Unaligned ends virus	Broken reads host	Disrupted genes	Nearby genes
14	N/A	9		BCL11B (-82287), SETD3 (+43263)

1. Identify Viral Integration Sites summary

Input reads	191,542
Host reads	39,544
Virus reads	151,161
Unmapped reads	837
Host reads (%)	20.65
Virus reads (%)	78.92
Unmapped reads (%)	0.44
Breakpoints identified	2
Viruses identified	2

2. Identify Viral Integration Sites virus content

Virus	Reads mapped
HPV16REF	75,785
HPV58REF	810

3. Identify Viral Integration Sites breakpoint summary

Host chromosome	Host region	Virus	Virus region	Unaligned ends host
10	complement (4607802..4607803)	HPV16REF	complement(2508.. 2509)	327
10	4768217..4768218	HPV16REF	2954..2955	111

Host chromosome	Unaligned ends virus	Broken reads host	Disrupted genes	Nearby genes
10	749	294		
10	416	130		AKR1E2 (+18411)

1. Identify Viral Integration Sites summary

Input reads	41,890
Host reads	15,941
Virus reads	25,613
Unmapped reads	336
Host reads (%)	38.05
Virus reads (%)	61.14
Unmapped reads (%)	0.80
Breakpoints identified	0
Viruses identified	4

2. Identify Viral Integration Sites virus content

Virus	Reads mapped
HPV51REF	8,464
HPV33REF	4,141
HPV16REF	264
HPV59REF	167

3. Identify Viral Integration Sites breakpoint summary

1. Identify Viral Integration Sites summary

Input reads	60,420
Host reads	24,520
Virus reads	35,464
Unmapped reads	436
Host reads (%)	40.58
Virus reads (%)	58.70
Unmapped reads (%)	0.72
Breakpoints identified	2
Viruses identified	3

2. Identify Viral Integration Sites virus content

Virus	Reads mapped
HPV16REF	17,414
HPV56REF	250
HPV58REF	221

3. Identify Viral Integration Sites breakpoint summary

Host chromosome	Host region	Virus	Virus region	Unaligned ends host
11	complement (62841861..62841862)	HPV16REF	complement(3251.. 3252)	66

S08_SRR8290163 (Report)

Host chromosome	Host region	Virus	Virus region	Unaligned ends host
11	62911538..62911539	HPV16REF	1255..1256	26

Host chromosome	Unaligned ends virus	Broken reads host	Disrupted genes	Nearby genes
11	206	57		ZBTB3 (-87677), POLR2G (-75151), TAF6L (-54519), TMEM223 (-49855), TMEM179B (-51461), NXF1 (-35559), STX5 (-9810), TEX54 (-9058), WDR74 (-52), SLC3A2 (+14142), CHRM1 (+66817), SLC22A6 (+94523)
11	159	21	CHRM1	STX5 (-79487), TEX54 (-78735), WDR74 (-69729), SLC3A2 (-22658), CHRM1, SLC22A6 (+24846), SLC22A8 (+77615)

1. Identify Viral Integration Sites summary

Input reads	188,058
Host reads	94,018
Virus reads	89,030
Unmapped reads	5,010
Host reads (%)	49.99
Virus reads (%)	47.34
Unmapped reads (%)	2.66
Breakpoints identified	5
Viruses identified	2

2. Identify Viral Integration Sites virus content

Virus	Reads mapped
HPV18REF	42,715
HPV16REF	2,571

3. Identify Viral Integration Sites breakpoint summary

Host chromosome	Host region	Virus	Virus region	Unaligned ends host
17	complement (64870507..64870508)	HPV18REF	complement(2398.. 2399)	59
17	46538811..46538812	HPV18REF	complement(2398.. 2399)	48
17	46320956..46320957	HPV18REF	complement(2398.. 2399)	67
17	47043099..47043100	HPV18REF	complement(2398.. 2399)	43
17	64867993..64867994	HPV18REF	2489..2490	277

Host chromosome	Unaligned ends virus	Broken reads host	Disrupted genes	Nearby genes
17	415	77	LRRC37A3	LRRC37A3
17	415	31	LRRC37A2, ARL17A	LRRC37A2, ARL17A, NSF (+51857)

S09_SRR8290160 (Report)

Host chromosome	Unaligned ends virus	Broken reads host	Disrupted genes	Nearby genes
17	415	51	ARL17B, LRRC37A	KANSL1 (-95567), ARL17B, LRRC37A
17	415	38	gene: ENSG00000262633	GOSR2 (-67575), gene: ENSG00000262633, RPRML (-63846), CDC27 (+74603)
17	275	210	LRRC37A3	LRRC37A3

1. Identify Viral Integration Sites summary

Input reads	295,226
Host reads	77,571
Virus reads	216,106
Unmapped reads	1,549
Host reads (%)	26.28
Virus reads (%)	73.20
Unmapped reads (%)	0.52
Breakpoints identified	2
Viruses identified	3

2. Identify Viral Integration Sites virus content

Virus	Reads mapped
HPV16REF	107,669
HPV90REF	1,757
HPV34REF	1,389

3. Identify Viral Integration Sites breakpoint summary

Host chromosome	Host region	Virus	Virus region	Unaligned ends host
8	complement (42250827..42250828)	HPV16REF	complement(6518.. 6519)	342
8	42250737..42250738	HPV16REF	2952..2953	106

Host chromosome	Unaligned ends virus	Broken reads host	Disrupted genes	Nearby genes
8	506	276		AP3M2 (-79154), PLAT (-43118), IKBKB (+20474), POLB (+87626)
8	302	138		AP3M2 (-79064), PLAT (-43028), IKBKB (+20564), POLB (+87716)

1. Identify Viral Integration Sites summary

Input reads	56,066
Host reads	14,544
Virus reads	6,903
Unmapped reads	34,619
Host reads (%)	25.94
Virus reads (%)	12.31
Unmapped reads (%)	61.75
Breakpoints identified	0
Viruses identified	5

2. Identify Viral Integration Sites virus content

Virus	Reads mapped
HPV16REF	2,875
HPV56REF	302
HPV58REF	199
HPV82REF	82
HPV33REF	44

3. Identify Viral Integration Sites breakpoint summary

1. Identify Viral Integration Sites summary

Input reads	10,914,512
Host reads	66,340
Virus reads	10,811,316
Unmapped reads	36,856
Host reads (%)	0.61
Virus reads (%)	99.05
Unmapped reads (%)	0.34
Breakpoints identified	3
Viruses identified	1

2. Identify Viral Integration Sites virus content

Virus	Reads mapped
HPV16REF	5,451,775

3. Identify Viral Integration Sites breakpoint summary

Host chromosome	Host region	Virus	Virus region	Unaligned ends host
1	complement (243110766.. 243110767)	HPV16REF	1874..1875	20
6	complement (106778509.. 106778510)	HPV16REF	4469..4470	19,647
6	106773522.. 106773523	HPV16REF	complement(6305.. 6306)	5,065

Host chromosome	Unaligned ends virus	Broken reads host	Disrupted genes	Nearby genes
1	N/A	16		CEP170 (+13661)
6	52,891	10,439		
6	26,387	4,349		

1. Identify Viral Integration Sites summary

Input reads	30,232
Host reads	22,356
Virus reads	7,594
Unmapped reads	282
Host reads (%)	73.95
Virus reads (%)	25.12
Unmapped reads (%)	0.93
Breakpoints identified	0
Viruses identified	5

2. Identify Viral Integration Sites virus content

Virus	Reads mapped
HPV18REF	3,131
HPV16REF	411
HPV33REF	161
HPV58REF	93
HPV56REF	61

3. Identify Viral Integration Sites breakpoint summary

1. Identify Viral Integration Sites summary

Input reads	1,771,566
Host reads	19,376
Virus reads	1,738,220
Unmapped reads	13,970
Host reads (%)	1.09
Virus reads (%)	98.12
Unmapped reads (%)	0.79
Breakpoints identified	10
Viruses identified	1

2. Identify Viral Integration Sites virus content

Virus	Reads mapped
HPV16REF	877,274

3. Identify Viral Integration Sites breakpoint summary

Host chromosome	Host region	Virus	Virus region	Unaligned ends host
3	complement (6423886..6423887)	HPV16REF	complement(4387.. 4388)	31
3	complement (6438439..6438440)	HPV16REF	6626..6627	82
3	6438431..6438432	HPV16REF	3255..3256	90
13	complement (73106941..73106942)	HPV16REF	3676..3677	21
13	complement (73052412..73052413)	HPV16REF	2296..2297	255
13	73088901..73088902	HPV16REF	complement(1972.. 1973)	206
13	73109252..73109253	HPV16REF	7236..7237	26
20	complement (30612243..30612244)	HPV16REF	4347..4348	394

S14_SRR8290154 (Report)

Host chromosome	Host region	Virus	Virus region	Unaligned ends host
20	complement (30629675..30629676)	HPV16REF	3950..3951	33
20	30612225..30612226	HPV16REF	complement(4387.. 4388)	1,541

Host chromosome	Unaligned ends virus	Broken reads host	Disrupted genes	Nearby genes
3	2,554	2		
3	N/A	53		
3	N/A	46		
13	N/A	11		PIBF1 (-90480), KLF5 (-29400)
13	1,182	243		PIBF1 (-35951), KLF5 (+2563)
13	740	140		PIBF1 (-72440), KLF5 (-11360)
13	N/A	13		PIBF1 (-92791), KLF5 (-31711)
20	1,808	276		
20	N/A	23		
20	2,554	646		

1. Identify Viral Integration Sites summary

Input reads	4,372,582
Host reads	34,716
Virus reads	4,321,432
Unmapped reads	16,434
Host reads (%)	0.79
Virus reads (%)	98.83
Unmapped reads (%)	0.38
Breakpoints identified	0
Viruses identified	2

2. Identify Viral Integration Sites virus content

Virus	Reads mapped
HPV58REF	2,116,408
HPV44REF	72,821

3. Identify Viral Integration Sites breakpoint summary

1. Identify Viral Integration Sites summary

Input reads	4,338,160
Host reads	79,839
Virus reads	4,244,375
Unmapped reads	13,946
Host reads (%)	1.84
Virus reads (%)	97.84
Unmapped reads (%)	0.32
Breakpoints identified	2
Viruses identified	3

2. Identify Viral Integration Sites virus content

Virus	Reads mapped
HPV90REF	1,166,607
HPV34REF	904,386
HPV51REF	79,784

3. Identify Viral Integration Sites breakpoint summary

Host chromosome	Host region	Virus	Virus region	Unaligned ends host
2	132427745..<br>132427746	HPV51REF	2321..2322	20
7	107770274..<br>107770275	HPV90REF	complement(6165..<br>6166)	21

Host chromosome	Unaligned ends virus	Broken reads host	Disrupted genes	Nearby genes
2	N/A	20	GPR39	GPR39
7	70	15	SLC26A3	SLC26A4 (-52465),<br>CBLL1 (-8607),<br>SLC26A3

1. Identify Viral Integration Sites summary

Input reads	2,699,196
Host reads	28,115
Virus reads	2,659,044
Unmapped reads	12,037
Host reads (%)	1.04
Virus reads (%)	98.51
Unmapped reads (%)	0.45
Breakpoints identified	29
Viruses identified	1

2. Identify Viral Integration Sites virus content

Virus	Reads mapped
HPV33REF	1,338,736

3. Identify Viral Integration Sites breakpoint summary

Host chromosome	Host region	Virus	Virus region	Unaligned ends host
1	complement (120735494.. 120735495)	HPV33REF	7499..7500	22
1	complement (120048063.. 120048064)	HPV33REF	7249..7250	51
1	complement (146207429.. 146207430)	HPV33REF	7249..7250	38
1	complement (148658168.. 148658169)	HPV33REF	7589..7590	50
1	120745535.. 120745536	HPV33REF	7249..7250	34
1	149412532.. 149412533	HPV33REF	7249..7250	32

S17_SRR8290151 (Report)

Host chromosome	Host region	Virus	Virus region	Unaligned ends host
1	120058091..120058092	HPV33REF	7499..7500	21
2	complement (212490303..212490304)	HPV33REF	2359..2360	25
2	complement (219219655..219219656)	HPV33REF	3101..3102	22
2	212406387..212406388	HPV33REF	6906..6907	20
2	240288737..240288738	HPV33REF	4242..4243	20
2	3590539..3590540	HPV33REF	4090..4091	21
3	134724804..134724805	HPV33REF	4729..4730	25
6	55381930..55381931	HPV33REF	4418..4419	48
10	complement (72834399..72834400)	HPV33REF	345..346	430
10	72856787..72856788	HPV33REF	2142..2143	20
10	72856788..72856789	HPV33REF	2142..2143	23
10	72856789..72856790	HPV33REF	2142..2143	28
10	72856791..72856792	HPV33REF	2142..2143	32
10	72856792..72856793	HPV33REF	2142..2143	31
10	72856812..72856813	HPV33REF	2143..2144	45
12	complement (131719612..131719613)	HPV33REF	4621..4622	23
12	131766235..131766236	HPV33REF	3885..3886	29
20	complement (50194454..50194455)	HPV33REF	4779..4780	34
X	complement (65927184..65927185)	HPV33REF	3516..3517	29
X	complement (65936859..65936860)	HPV33REF	1168..1169	48
X	65937208..65937209	HPV33REF	400..401	27
X	65936851..65936852	HPV33REF	3509..3510	23
X	65927164..65927165	HPV33REF	2871..2872	45

Host chromosome	Unaligned ends virus	Broken reads host	Disrupted genes	Nearby genes
1	N/A	9	NOTCH2NLR, NBPF26	NOTCH2NLR, NBPF26

S17_SRR8290151 (Report)

Host chromosome	Unaligned ends virus	Broken reads host	Disrupted genes	Nearby genes
1	N/A	13	NOTCH2	NOTCH2
1	N/A	9	NBPF10, NOTCH2NLA	NBPF10, NOTCH2NLA
1	N/A	8	NBPF14, NOTCH2NLB	NBPF14, NOTCH2NLB, NUDT4B (+90605)
1	N/A	9	NOTCH2NLR, NBPF26	NOTCH2NLR, NBPF26
1	N/A	13	NOTCH2NLC, gene: ENSG00000286185	NOTCH2NLC, gene: ENSG00000286185, NBPF19 (+62512)
1	N/A	10	NOTCH2	NOTCH2, SEC22B (+92806)
2	N/A	4	ERBB4	ERBB4
2	N/A	7	gene: ENSG00000284820, ATG9A	NHEJ1 (-58790), gene: ENSG00000280537 (-48828), SLC23A3 (-49560), CNPPD1 (-41549), RETREG2 (-34180), ZFAND2B (-10004), ABCB6 (-661), gene: ENSG00000284820, ATG9A, ANKZF1 (+10127), GLB1L (+16942), STK16 (+25799), TUBA4A (+30054), TUBA4B (+33587), DNAJB2 (+59686), PTPRN (+69967)
2	N/A	16	ERBB4	ERBB4
2	N/A	5		
2	N/A	9		ADI1 (-71008), gene: ENSG00000286905 (-32206), RNASEH1 (-32206), RPS7 (-9619), COLEC11 (+4292), ALLC (+67660)
3	N/A	2		KY (-73168), EPHB1 (+70455)
6	N/A	17	GFRAL	HCRTR2 (-99313), GFRAL, HMGCLL1 (+52442)
10	1,074	175	MCU	MCU, gene: ENSG00000279502 (-77383), OIT3 (+59184)

S17_SRR8290151 (Report)

Host chromosome	Unaligned ends virus	Broken reads host	Disrupted genes	Nearby genes
10	N/A	120	MCU	MCU, gene: ENSG00000279502 (-99771), OIT3 (+36796), PLA2G12B (+77974)
10	N/A	134	MCU	MCU, gene: ENSG00000279502 (-99772), OIT3 (+36795), PLA2G12B (+77973)
10	N/A	147	MCU	MCU, gene: ENSG00000279502 (-99773), OIT3 (+36794), PLA2G12B (+77972)
10	N/A	148	MCU	MCU, gene: ENSG00000279502 (-99775), OIT3 (+36792), PLA2G12B (+77970)
10	N/A	148	MCU	MCU, gene: ENSG00000279502 (-99776), OIT3 (+36791), PLA2G12B (+77969)
10	N/A	192	MCU	MCU, gene: ENSG00000279502 (-99796), OIT3 (+36771), PLA2G12B (+77949)
12	N/A	16	SFSWAP	gene: ENSG00000279455 (-98847), gene: ENSG00000279153 (-73168), SFSWAP
12	N/A	15	SFSWAP	SFSWAP, MMP17 (+62157)
20	N/A	14		UBE2V1 (-78495), PEDS1-UBE2V1 (-40817), PEDS1 (-40720), CEBPB (-1786)
X	N/A	23		VSIG4 (+94553)
X	N/A	30		VSIG4 (+84878)
X	N/A	39		VSIG4 (+84529)
X	N/A	9		VSIG4 (+84886)

S17_SRR8290151 (Report)

Host chromosome	Unaligned ends virus	Broken reads host	Disrupted genes	Nearby genes
X	N/A	17		VSIG4 (+94573)

1. Identify Viral Integration Sites summary

Input reads	70,954
Host reads	23,410
Virus reads	46,400
Unmapped reads	1,144
Host reads (%)	32.99
Virus reads (%)	65.39
Unmapped reads (%)	1.61
Breakpoints identified	1
Viruses identified	2

2. Identify Viral Integration Sites virus content

Virus	Reads mapped
HPV16REF	20,660
HPV33REF	2,803

3. Identify Viral Integration Sites breakpoint summary

Host chromosome	Host region	Virus	Virus region	Unaligned ends host
8	complement (24153941..24153942)	HPV16REF	complement(3520.. 3521)	121

Host chromosome	Unaligned ends virus	Broken reads host	Disrupted genes	Nearby genes
8	241	87		

1. Identify Viral Integration Sites summary

Input reads	160,780
Host reads	21,846
Virus reads	134,589
Unmapped reads	4,345
Host reads (%)	13.59
Virus reads (%)	83.71
Unmapped reads (%)	2.70
Breakpoints identified	2
Viruses identified	6

2. Identify Viral Integration Sites virus content

Virus	Reads mapped
HPV58REF	40,336
HPV18REF	12,653
HPV33REF	10,228
HPV68REF	2,740
HPV44REF	1,450
HPV59REF	402

3. Identify Viral Integration Sites breakpoint summary

Host chromosome	Host region	Virus	Virus region	Unaligned ends host
21	complement (41928658..41928659)	HPV18REF	complement(4548.. 4549)	23
21	41928655..41928656	HPV18REF	3519..3520	47

Host chromosome	Unaligned ends virus	Broken reads host	Disrupted genes	Nearby genes
21	157	27	C2CD2	PRDM15 (-49176), C2CD2, ZBTB21 (+58172)

S19_SRR8290149 (Report)

Host chromosome	Unaligned ends virus	Broken reads host	Disrupted genes	Nearby genes
21	141	30	C2CD2	PRDM15 (-49173), C2CD2, ZBTB21 (+58175)

1. Identify Viral Integration Sites summary

Input reads	234,806
Host reads	109,996
Virus reads	120,053
Unmapped reads	4,757
Host reads (%)	46.85
Virus reads (%)	51.13
Unmapped reads (%)	2.03
Breakpoints identified	2
Viruses identified	1

2. Identify Viral Integration Sites virus content

Virus	Reads mapped
HPV18REF	60,681

3. Identify Viral Integration Sites breakpoint summary

Host chromosome	Host region	Virus	Virus region	Unaligned ends host
22	complement (40756238..40756239)	HPV18REF	complement(1962.. 1963)	98

S20_SRR8290148 (Report)

Host chromosome	Host region	Virus	Virus region	Unaligned ends host
22	40755123..40755124	HPV18REF	1396..1397	146

Host chromosome	Unaligned ends virus	Broken reads host	Disrupted genes	Nearby genes
22	360	87		TNRC6B-201 (-432997), TNRC6B-203 (-432997), TNRC6B-202 (-432997), TNRC6B-206 (-432997), TNRC6B-205 (-435005), CDS: ENSP00000492828 (-406381), ADSL-215 (-406176), ADSL-209 (-397282), ADSL-226 (-397282), ADSL-214 (-397282), ADSL-206 (-395121), ADSL-232 (-395121), ADSL-218 (-391876), ADSL-211 (-389716), ADSL-202 (-389716), ADSL-228 (-389716), ADSL-208 (-389716), ADSL-216 (-389716), ADSL-201 (-389716), ADSL-231 (-389716), ADSL-221 (-388620), ADSL-233 (-388620), ADSL-222 (-382881), ADSL-220 (-369008), ADSL-212 (-394672), ADSL-213 (-388620), ADSL-203 (-388620), ADSL-207 (-365925), SGSM3-201 (-346479), SGSM3-204 (-350015), SGSM3-203 (-346581), SGSM3-202 (-346479), MRTFA-211 (-344591), MRTFA-212 (-319928), MRTFA-205 (-293011), MRTFA-217 (-222603), MRTFA-203 (-203892), MRTFA-201 (-203892), MRTFA-210 (-319928), MRTFA-202 (-293011), MRTFA-215 (-203892), MRTFA-204 (-293011), MRTFA-216 (-324818), MRTFA-206 (-236753), MRTFA-218 (-264041), MRTFA-207 (-203892), MCHR1-201 (-74310), MCHR1-202 (-74310), SLC25A17-202 (+14595), SLC25A17-217 (+14595), SLC25A17-210

S20_SRR8290148 (Report)

Host chromosome	Unaligned ends virus	Broken reads host	Disrupted genes	Nearby genes
22	692	146		TNRC6B-201 (-431882), TNRC6B-203 (-431882), TNRC6B-202 (-431882), TNRC6B-206 (-431882), TNRC6B-205 (-433890), CDS: ENSP00000492828 (-405266), ADSL-215 (-405061), ADSL-209 (-396167), ADSL-226 (-396167), ADSL-214 (-396167), ADSL-206 (-394006), ADSL-232 (-394006), ADSL-218 (-390761), ADSL-211 (-388601), ADSL-202 (-388601), ADSL-228 (-388601), ADSL-208 (-388601), ADSL-216 (-388601), ADSL-201 (-388601), ADSL-231 (-388601), ADSL-221 (-387505), ADSL-233 (-387505), ADSL-222 (-381766), ADSL-220 (-367893), ADSL-212 (-393557), ADSL-213 (-387505), ADSL-203 (-387505), ADSL-207 (-364810), SGSM3-201 (-345364), SGSM3-204 (-348900), SGSM3-203 (-345466), SGSM3-202 (-345364), MRTFA-211 (-343476), MRTFA-212 (-318813), MRTFA-205 (-291896), MRTFA-217 (-221488), MRTFA-203 (-202777), MRTFA-201 (-202777), MRTFA-210 (-318813), MRTFA-202 (-291896), MRTFA-215 (-202777), MRTFA-204 (-291896), MRTFA-216 (-323703), MRTFA-206 (-235638), MRTFA-218 (-262926), MRTFA-207 (-202777), MCHR1-201 (-73195), MCHR1-202 (-73195), SLC25A17-202 (+15710), SLC25A17-217 (+15710), SLC25A17-210 (+15710), SLC25A17-204 (+21158), SLC25A17-205 (+23981), SLC25A17-

1. Identify Viral Integration Sites summary

Input reads	31,718
Host reads	26,475
Virus reads	4,978
Unmapped reads	265
Host reads (%)	83.47
Virus reads (%)	15.69
Unmapped reads (%)	0.84
Breakpoints identified	0
Viruses identified	5

2. Identify Viral Integration Sites virus content

Virus	Reads mapped
HPV16REF	2,102
HPV58REF	214
HPV56REF	122
HPV33REF	41
HPV82REF	35

3. Identify Viral Integration Sites breakpoint summary
